# MAGL targeted PROTAC degrader simultaneously enhances P53 for synergistic treatment of glioblastoma stem cell

**DOI:** 10.1038/s41420-025-02392-1

**Published:** 2025-03-20

**Authors:** Zheng Yuan, Meixia Guo, Yue Zhang, Yilin Deng, Biao Sun, Yaning Hou, Xin Wang, Xiong Jin, Yang Liu, Bingyang Shi, Jinlong Yin

**Affiliations:** 1 Henan Key Laboratory of Brain Targeted Bio-nanomedicine, School of Life Sciences, Henan University, Kaifeng, Henan China; 2 Henan-Macquarie University Joint Centre for Biomedical Innovation, School of Life Sciences, Henan University, Kaifeng, Henan China; 3 Department of Cancer Biomedical Science, Graduate School of Cancer Science and Policy, National Cancer Center, Goyang, Republic of Korea; 4 Huaihe Hospital of Henan University, Kaifeng, China

**Keywords:** CNS cancer, Targeted therapies

## Abstract

Glioblastoma (GBM) stands as the most fatal brain tumor due to limited therapeutic options and high rates of drug resistance. Current surgical and pharmacological interventions usually fail to eradicate the aggressive GBM stem cells (GSCs), which leads to the deadly GBM occurrence. Although proteolysis-targeting chimeras (PROTACs) are prosperous in drug development for tumors, their application in GBM, particularly for GSC-sensitive drug candidates remains in its nascent stages. In this regard, we designed a monoacylglycerol lipase (MAGL) targeting PROTAC, where MAGL was identified as a novel target for GSCs in our previous study. The MAGL inhibitor JZL184 was redesigned by leveraging computational chemistry analysis, and an active unit was engaged for conjugation. E3 ligand for MAGL targeted warhead conjugation was screened with bioinformatics analyses, which revealed heightened activity of the E3 ligase MDM2 in GBM, a classic negative regulator of the tumor suppressor P53, which correlates with patient prognosis. Then the PROTAC was conjugated with JZL184 analog and the MDM2 inhibitor Nutlin-3 analog. Experimental results validated that the designed JN-PROTAC effectively induced MAGL targeted degradation and concomitantly enhanced P53 activation via MDM2 inhibition and is capable of inhibiting the progression of patient-derived GSCs in vivo. This work presents a proof-of-concept PROTAC design tailored for GSCs, potentially addressing the occurrence challenges for GBM.

## Introduction

Glioblastoma (GBM) is the most common and lethal brain tumor with a critically low survival rate, the median survival period of GBM patients only remains 14 months, and 2-year survival rates <10% [1–3]. The development of anti-GBM drugs has been with great difficulties reflected by lipophilic alkylating agent Temozolomide (TMZ) stands as the only clinically available oral administrated GBM drug for decades [4]. GBM is highly invasive and prone to acquiring TMZ resistance due to its high mutation rate and limited treatment options. TMZ primarily damages DNA through alkylation or methylation mechanism, rather than specifically targeting the pathways involved in GBM progression [5, 6]. TMZ-induced DNA repair deficiencies frequently lead to genomic instability, which can accelerate the progression from low-grade glioma to GBM and promote tumor recurrence [7]. The lethality and burden of GBM can also be attributed to insufficient clearance of GBM stem cells (GSCs) [8], leading to high recurrence rates and drug resistance [9]. Therefore, there is a critical need for therapeutic agents that precisely target GSCs.

PROTACs (proteolysis-targeting chimeras) are emerging as a promising therapeutic strategy for targeted protein degradation (TPD). PROTACs are designed to selectively degrade a protein of interest (POI) by recruiting an E3 ubiquitin ligase, which facilitates the ubiquitination of the POI [10]. This process ultimately leads to the degradation of the POI via the proteasome system [11]. PROTACs are generally composed of heterobifunctional molecules that simultaneously bind to both the POI and an E3 ligase. Unlike traditional small-molecule inhibitors, which primarily function by blocking protein activity through occupancy pharmacology, PROTACs have the ability to eliminate the target protein entirely. This mechanism offers the potential for improved therapeutic outcomes [10]. The choice of E3 ligase is crucial for improving the specificity and reducing the toxicity of PROTACs [12]. For PROTAC development, von Hippel-Lindau (VHL) and cereblon (CRBN) are the most commonly utilized E3 ligases [13]. Utilizing VHL ligands, which are expressed at low levels in platelets, can help reduce blood toxicity, thereby improving the drug safety profile [14].

In our previous study, monoacylglycerol lipase (MAGL) was identified as a key player in the regulation of the self-renewal and tumorigenicity of GSCs, as well as in connecting the interplay between GSCs and tumor-associated macrophages [15]. GSCs have emerged as a critical target for GBM due to their crucial role in drug resistance and high occurrence rate [16–18]. However, current therapeutics targeting GSCs remain very limited due to their intra-tumor heterogeneity [19]. MAGL, as a promising target candidate [20, 21], functions as a key enzyme that catalyzes the breakdown of glycerol monoesters of long-chain fatty acids, regulating intracellular triglyceride stores and the metabolism of fatty acids and glycerol in adipocytes and other cells [22]. The MAGL small-molecule inhibitor JZL184 was applied to a patient-derived GSC xenograft model and increased the survival rate [15], however, the specificity issue of JZL184 requires further improvement. Given that MAGL has physiological functions in various tissues, its broader application in anti-GBM therapies may be limited by off-target effects. To address these specificity challenges, the PROTAC strategy offers a valuable solution. By conjugating MAGL inhibitors with various ligands, PROTACs can enhance tissue specificity in areas where the corresponding E3 ligase is abundant, effectively minimizing off-target effects and increasing the therapeutic potential of MAGL inhibition. In this context, a MAGL-targeting PROTAC is proposed as an innovative approach to enhance tumor specificity while reducing toxicity. To optimize MAGL inhibitors for GBM therapy, rational drug design is essential to ensure greater specificity and reduced systemic toxicity. Utilizing PROTACs may help overcome these challenges and improve therapeutic outcomes in GBM treatment.

In this study, by analyzing the clinical metadata, ligands for MDM2 E3 ligase were proposed for the PROTAC design, due to GBM expresses a relatively high level of MDM2. Moreover, pioneers in PROTAC, Dr. Crews’s team, also proved the MDM2 recruiting PROTAC may offer synergistic antitumor activity by simultaneously degrading the POI and stabilizing the P53 [23]. All these points suggest the feasibility of the designed MAGL-targeting PROTAC. To facilitate PROTAC synthesis, we first analyzed the binding model of the MAGL small molecule inhibitor JZL184 and designed the MAGL-targeting warhead based on molecular docking data. Next, the new JZL184 analog was linked with a Nutlin analog for MDM2 targeting. The designed PROTAC was then proposed and synthesized for further evaluation of its anti-tumor capacity.

## Results

### Computer-aided rational design for PROTAC molecule warhead and ligand

**Fig. 1:**
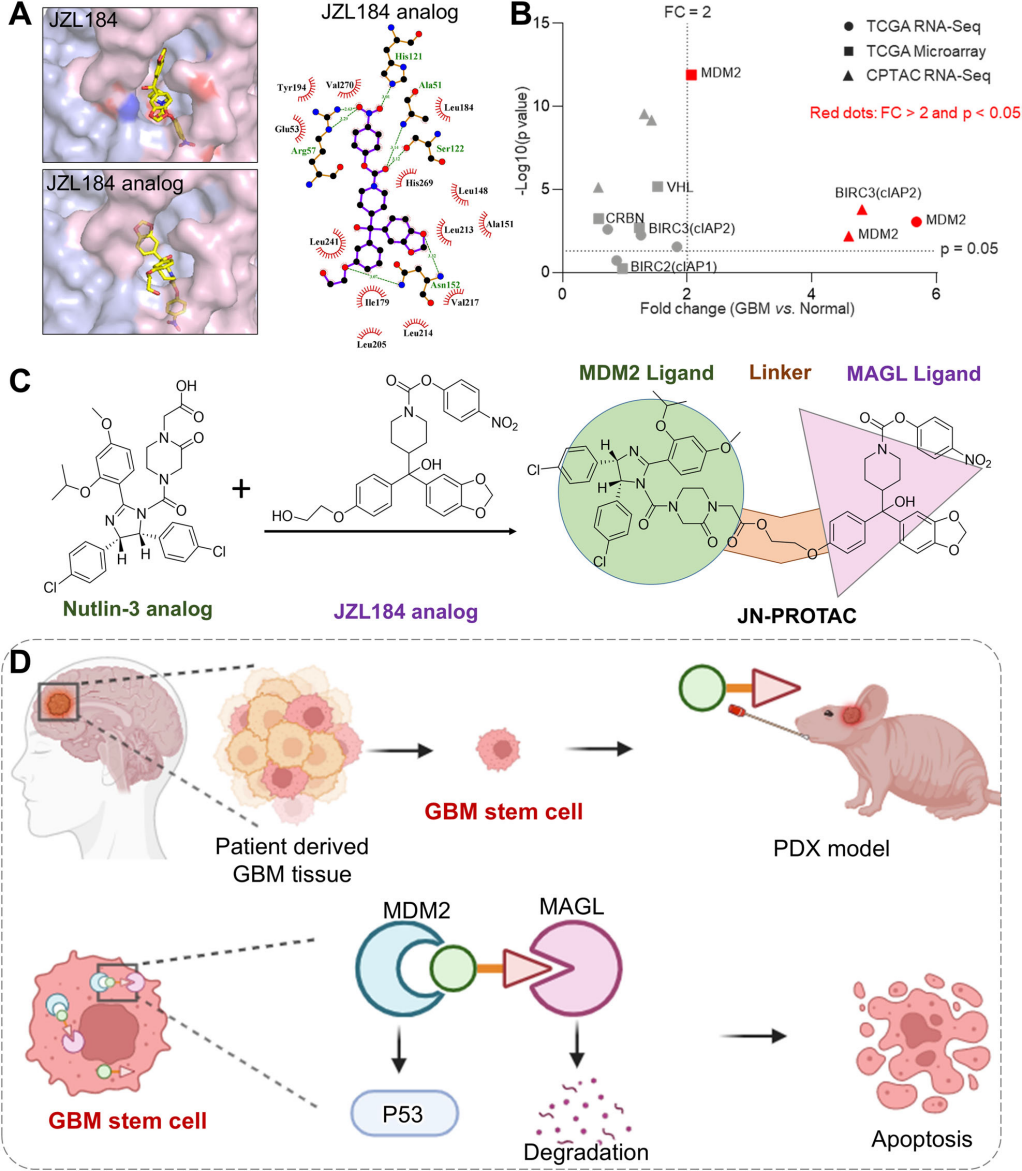
Computer-aided rational design and working model for JN-PROTAC. **A** Molecular docking of the MAGL protein (PDB ID: 3HJU) with the commercial inhibitor JZL184 (left) and the designed JZL184 analog for synthesis (right). The docking results show the binding models and the interactions of the amino acid residues with the designed JZL184 analog (right panel). **B** Clinical metadata screening for glioblastoma E3 ligase. Data are collected from RNAseq and microarray datasets of TCGA and CPTAC databases. The X-axis is fold changes of general E3 ligases in GBM vs. Normal tissue. The Y-axis is the *p*-values presented as –Log10 (*p*-value). Candidates with fold changes (FC) > 2 and *p* < 0.05 were noted in red. E3 ligase MDM2 is highly expressed in GBM. **C** Chemical structure design of JN-PROTAC, which links JZL184 analog and Nutlin-3 analog. **D** Conceptual scheme of JN-PROTAC evaluation in this study.

The investigation was initiated with the computer-aided rational design for the PROTAC molecule warhead and ligand. Inspired by the molecular docking results of MAGL protein to its corresponding small molecular inhibitor JZL184 (Supplementary Fig. 1), the piperonyl groups of JZL184 were identified as having less interaction with the MAGL protein, which indicates a JZL184 analog with piperonyl ring-open and replacing with the active group may be a suitable warhead for MAGL targeting ligand conjugation. Therefore, the JZL184 analog was designed and synthesized for this PROTAC (Fig. 1A and Supplementary Fig. 1). We further screened the E3 ligase expression in GBM through bioinformatics analysis with the metadata in the clinical database TCGA to guide the E3 ligase selection. Data mining from TCGA RNA-seq, microarray, and CPTAC RNA-Seq together indicate the highly expressed MDM2 in GBM could be a potential ligase (Fig. 1B). Furthermore, we verified MDM2 expression using proteomics databases, patient-derived tumor and adjacent tissues, as well as tumor and normal cell lines. The data demonstrated that MDM2 expression is enriched in tumor cells compared to normal tissues, which may enhance the specificity of this PROTAC (Supplementary Fig. 2). Conjugating the ligand of MDM2 to the designed MAGL warhead may enhance the specificity of the PROTAC and consequently reduce the off-target toxicity. To this regard, MDM2 corresponding small molecular inhibitor Nutlin-3 was designed into this MAGL targeting PROTAC. We crosslinked the COOH group in the Nutlin-3 analog to the OH group in the JZL184 analog and obtained the designed JN-PROTAC (Fig. 1C and Supplementary Information). The synthesis routine and characterization of JN-PROTAC are shown in Supplementary Figs. 3-6. Then the performance evaluation of the JN-PROTAC was assessed both in vitro and in vivo (Fig. 1D).

### JN-PROTAC induces MAGL targeted degradation in multiple tumor cells and enhances P53 expression

**Fig. 2:**
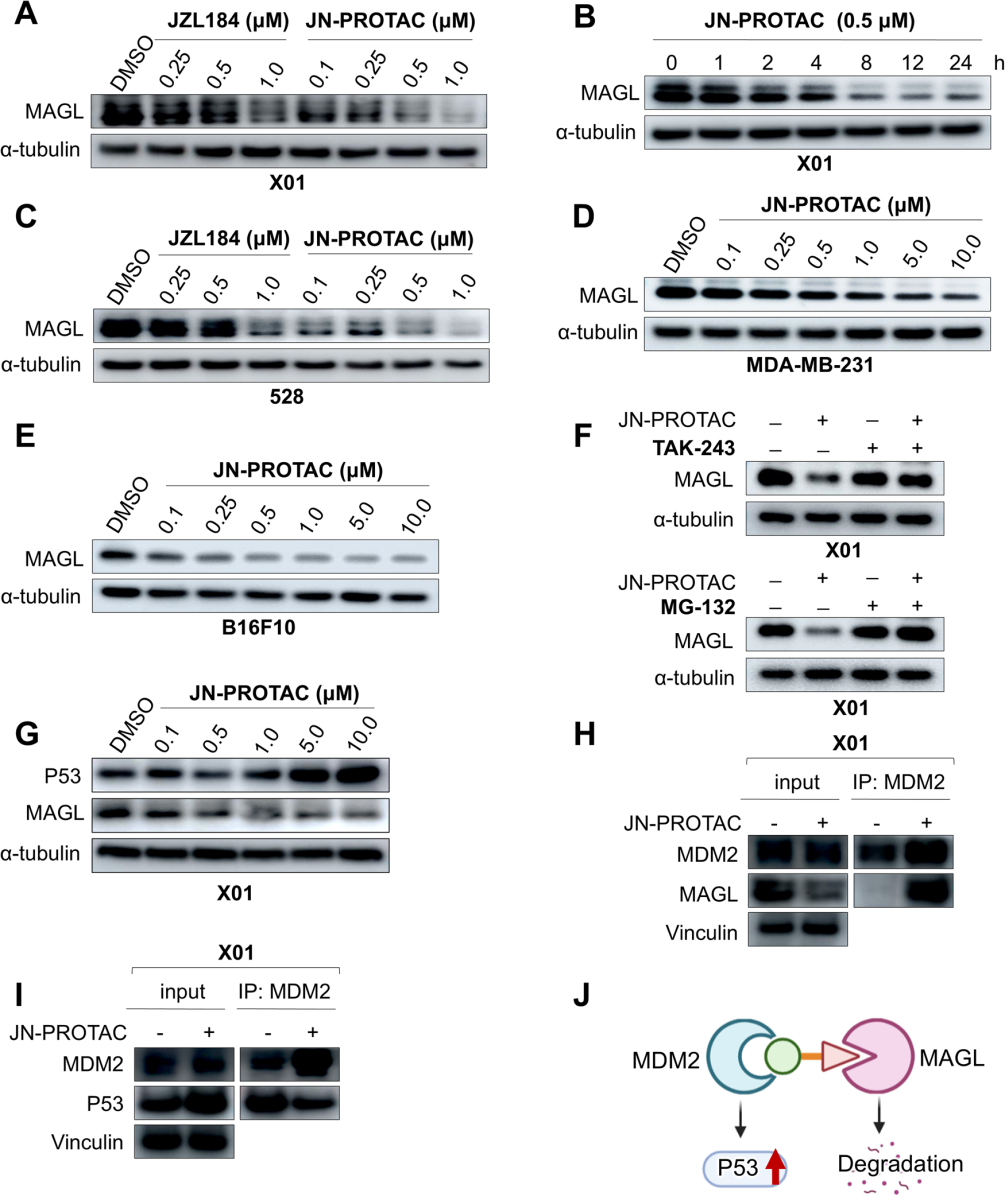
JN-PROTAC induces MAGL-targeted degradation in multiple tumor cells and enhances P53 expression. **A** Immunoblotting for MAGL expression in X01 GSCs after 24 h treatment with the indicated concentrations of JZL184 or JN-PROTAC. α-tubulin was used as a loading control. **B** Immunoblotting for MAGL expression in X01 GSCs treated with 0.5 μM of JN-PROTAC for the indicated durations. **C** Immunoblotting for MAGL expression in 528 GSCs after 24 h treatment with the indicated concentrations of JZL184 or JN-PROTAC. **D**, **E** Immunoblotting for MAGL expression in human breast tumor MDA-MB-231 cells (**D**) and murine melanoma B16F10 cells (**E**) after 24 h treatment with the indicated concentrations of JN-PROTAC. **F** Immunoblotting for MAGL expression in X01 GSCs after 24 h treatment with 500 nM of JN-PROTAC, 10 μM of TAK-243 or 10 μM of MG-132. TAK-243 and MG-132 are UBA1 inhibitor and proteasome inhibitor, respectively. **G** Immunoblotting for P53 and MAGL expression in X01 cells after 24 h treatment with the indicated concentrations of JN-PROTAC. **H** Co-IP assay for the PROTAC-induced ternary complex formation in X01 GSCs treated with JN-PROTAC (10 μM) or DMSO for 48 h. Cell lysates were precipitated with anti-MDM2 antibody. Vinculin was used as a loading control. **I** Co-IP assay for the interaction of MDM2 and P53 in X01 GSCs treated with JN-PROTAC (10 μM) or DMSO for 48 h. Cell lysates were precipitated with anti-MDM2 antibody. Vinculin was used as a loading control. **J** The proposed dual functional role of the JN-PROTAC in MAGL degradation and activation of tumor suppressor P53 through MDM2 blocking.

Following the rational synthesis of MDM2-recruited JN-PROTAC, we firstly tested the hypothesis that JN-PROTAC can mediate MAGL degradation and simultaneously enhance tumor suppressor P53 expression in response to the MDM2 inhibition in well-established X01 GSCs. Data suggest a dose dependent MAGL degradation was triggered compared with the JZL184 treatment alone group, and the functional clearance of MAGL protein can reach less than 1 μM in X01 and 528 GSCs (Fig. 2A, C). Moreover, the MAGL degradation can be observed as early as 8 h post-treatment, which indicates the functional MAGL turnover by JN-PROTAC is rapid and sufficient (Fig. 2B and Supplementary Fig. 7A). The hook effect may occur when high concentrations of a PROTAC lead to a reduction in its efficacy, often due to the saturation of target binding to one side, rather than forming the classical ternary complex of the PROTAC [24–26]. This phenomenon is typically observed at higher doses and can result in diminished activity despite increasing the compound’s concentration. In this study, JN-PROTAC exhibited a classical hook effect at concentrations of 50 μM or higher, reflecting its mechanism of action (Supplementary Fig. 7B, C). Next, we moved to investigate the antitumor potential of this JN-PROTAC and answer whether it can work in different tumor cells. To this regard, MDA-MB-231 human breast tumor cells and mouse melanoma B16F10 cells were tested and a dose-dependent MAGL degradation was shown (Fig. 2D, E). To validate the JN-PROTAC-mediated MAGL protein degradation through the typical proteosome-mediated protein recycling pathway, UBA1 (ubiquitin-activating enzyme 1) inhibitor TAK-243 and proteasome inhibitor MG-132 were used for degradation pathway blocking in X01 and 528 GSCs [27, 28], which is in line with the typical PROTAC mechanism (Fig. 2F and Supplementary Fig. 7D, E). Considering the Nutlin-3 analog was designed into this MAGL-PROTAC for E3 ligase MDM2 binding, activation of P53 may occur as a consequence under the JN-PROTAC treatment. Data indeed showed a dose dependent P53 activation in X01 GSCs (Fig. 2G), which confirmed the advantage of P53/MAGL dual targeting PROTAC and may offer a better GSCs inhibition outcome. To determine PROTAC-induced ternary complex formation, we conducted an immunoprecipitation assay. The data demonstrated the binding of MDM2 to MAGL, reflecting the bridging function of JN-PROTAC (Fig. 2H and Supplementary Fig. 7F). Moreover, the co-IP data demonstrated a slight decrease in MDM2-P53 interaction upon PROTAC treatment. This may be attributed to the ability of JN-PROTAC to bind the MDM2 for targeted degradation, thereby limiting the binding capacity between MDM2 and P53. This interaction reduction may enhance P53 expression, contributing to tumor suppression (Fig. 2I and Supplementary Fig. 7G). This further supports the synthesized JN-PROTAC effectively targets MAGL for degradation while simultaneously modulating the MDM2-P53 interaction. Notably, in the P53-mutant GBM cell line U251 [29], JN-PROTAC exhibited anti-tumor activity through MAGL degradation (Supplementary Fig. 7H), indicating that MAGL degradation can promote cell death independently of P53-mediated pathways (Fig. 2J). Proteomics analysis was conducted to further investigate the proteomic profile under JN-PROTAC treatment. Functional annotation of differentially expressed proteins (DEPs) revealed fatty acid metabolism as the most significantly associated pathway, which aligns with the well-established role of MAGL [22] (Supplementary Fig. 8).

### JN-PROTAC triggers the GSCs proliferation arrest and apoptosis

**Fig. 3:**
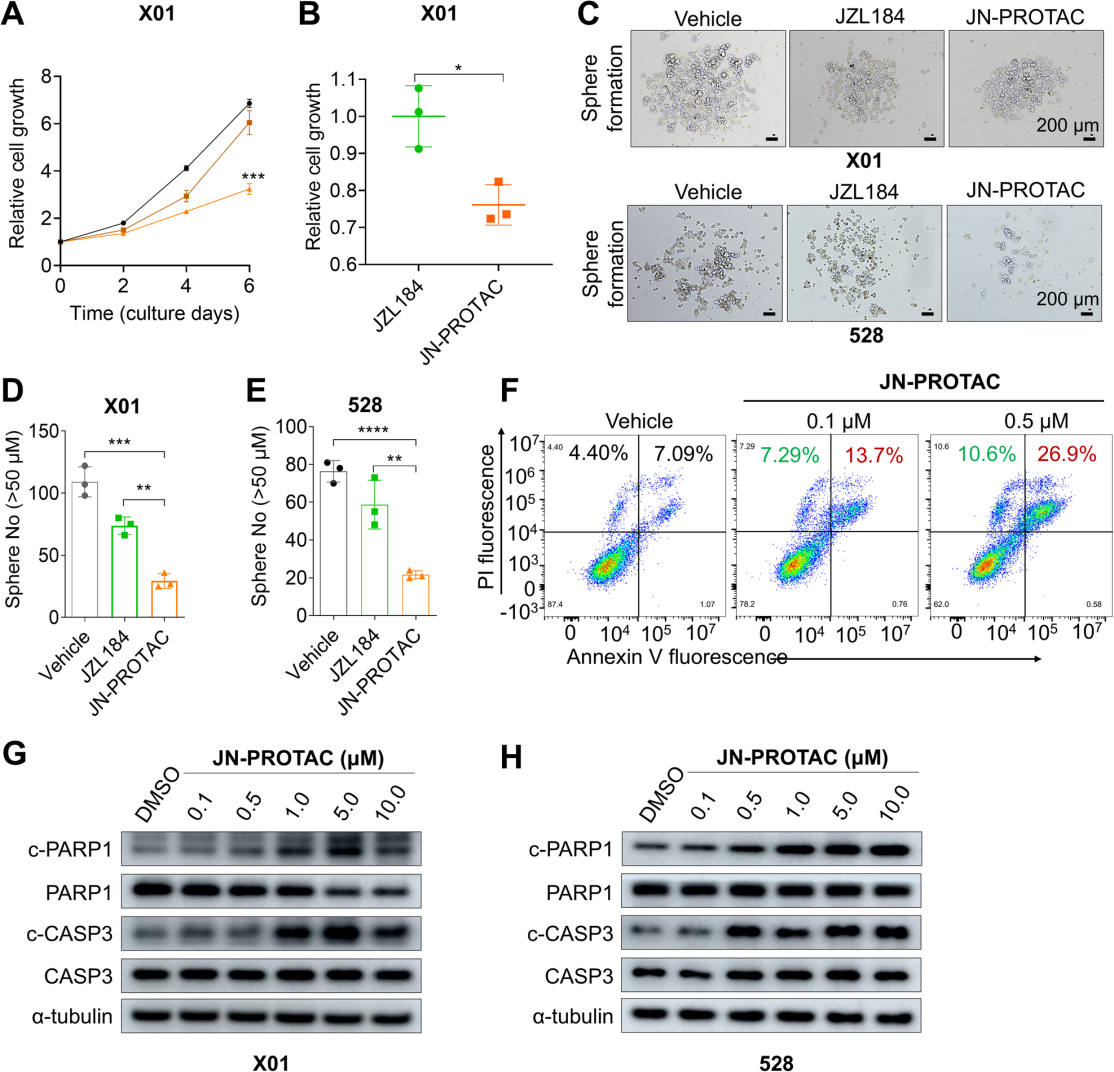
JN-PROTAC effectively inhibits GSC proliferation and induces apoptosis. **A** Cell proliferation assays were performed in X01 GSCs treated with JN-PROTAC (0.1 μM brown and 1 μM orange) for 6 days. Relative proliferation folds data are presented as mean ± SD (*n* = 3, independent experiments), two-tailed Student’s t-test. **B** The growth inhibitory assay of JN-PROTAC (1 μM) and JZL184 (1 μM) was assessed in X01 GSCs. Cells were treated for 6 days and compared with the average cell number of the JZL184 treatment group. **C** Representative images for sphere-formation assays performed using GSCs (X01 and 528) treated with 0.5 μM of JZL184, 0.5 μM of JN-PROTAC, or vehicle, respectively. Images are representative of three independent experiments. Scale bar, 200 µm. **D**, **E** Quantification for the average number of spheres with a diameter over 50 μm. **F** Flow cytometry for PI/Annexin-V apoptosis assay in X01 GSCs treated with JN-PROTAC (0.1 μM and 0.5 μM). **G**, **H** Immunoblotting for cleaved-PARP1 (c-PAPR1), PARP1, cleaved-Caspase3 (c-CASP3), and Caspase3 (CASP3) expression in X01 (**G**) and 528 GSCs (**H**) after 8 h treatment with the indicated concentrations of JN-PROTAC. α-tubulin was used as a loading control. All quantification data are presented as mean ± SD (*n* = 3, independent experiments), two-tailed Student’s t-test (*p < 0.05, **p < 0.01, ***p < 0.001).

To demonstrate the dose-dependent efficacy of JN-PROTAC, we conducted a quantitative analysis of immunoblotting data to evaluate MAGL degradation in 528 and X01 GSCs following treatment with varying concentrations of JN-PROTAC. The DC_50_, defined as the concentration at which 50% of the maximum effect is observed, was calculated for MAGL degradation in 528 GSCs to be 60.28 nM, with a corresponding D_max_, representing the maximum degradation achieved, of approximately 95% (Supplementary Fig. 9A). In X01 GSCs, the DC_50_ was determined to be 373.6 nM, with a D_max_ of approximately 92.95% (Supplementary Fig. 9B). Notably, previous studies utilizing immunoblotting have also reported similar findings [30, 31]. Future studies could employ the more advanced HiBiT lytic assay to further assess the dose-response of the PROTAC. We then assessed the cellular effects of JN-PROTAC. GSCs are highly proliferating self-renewal cells and exhibit typical stem cell characteristics such as sphere-forming ability, reflecting the stemness. To this regard, we first tested the cell growth by the optical intensity of the living floating cells in a culture medium, data showed a dose-dependent growth arrest of the JN-PROTAC treated X01 GSCs and significantly decreased the GSC proliferation at day 6 post treatments compared with JZL184 treatment group (Fig. 3A, B). To validate the specificity of JN-PROTAC for MAGL-expressing tumor cells, we assessed MAGL expression across various cell lines, including normal human astrocytes, GBM stem cells, mouse melanoma (B16F10), and human breast tumor (MDA-MB-231) cells. The data indicated that all tested tumor cell lines exhibited higher levels of MAGL (Supplementary Fig. 9C). Notably, JN-PROTAC did not significantly inhibit growth in normal astrocytes (Supplementary Fig. 9D), likely due to the low levels of MDM2 and MAGL expression in normal astrocytes. Furthermore, GSC sphere-formation capacity was evaluated, and representative images of the X01 and 528 GSCs clones were presented (Fig. 3C). Quantification data also demonstrated the JN-PROTAC is more powerful than JZL184 treatment in inhibiting the GSC sphere clone formation (Fig. 3D, E), which supports the hypothesis that JN-PROTAC upgraded from JZL184 combined the MAGL targeted degradation and P53 activation is more powerful and may be capable for GSCs in vivo treatment.

As the GSC growth inhibition was observed, we further wondered whether the inhibition was associated with programmed cell death of the GSCs. To evaluate this, flow cytometry analysis for Annexin-V and PI co-staining was applied and confirmed the X01 GSCs death in a dose-dependent manner (Fig. 3F). To evaluate whether apoptosis exhibited a concentration-dependent pattern, we extended our analysis to higher JN-PROTAC concentrations, ranging from 0.5 μM to 5 μM. The results showed an increase in the apoptotic cell population as the concentration of JN-PROTAC increased in both GSCs (Supplementary Fig. 9E-H). The apoptosis initiation was also confirmed with the core apoptosis effect factors cleaved-PARP1 and cleaved-Caspase3 in X01 and 528 GSCs (Fig. 3G, H). Collectively, these data demonstrated a better anti-GBM ability of JN-PROTAC compared with JZL184 and may further be available in vivo.

### In vivo antitumor potential of JN-PROTAC in X01 GSCs PDX model

**Fig. 4:**
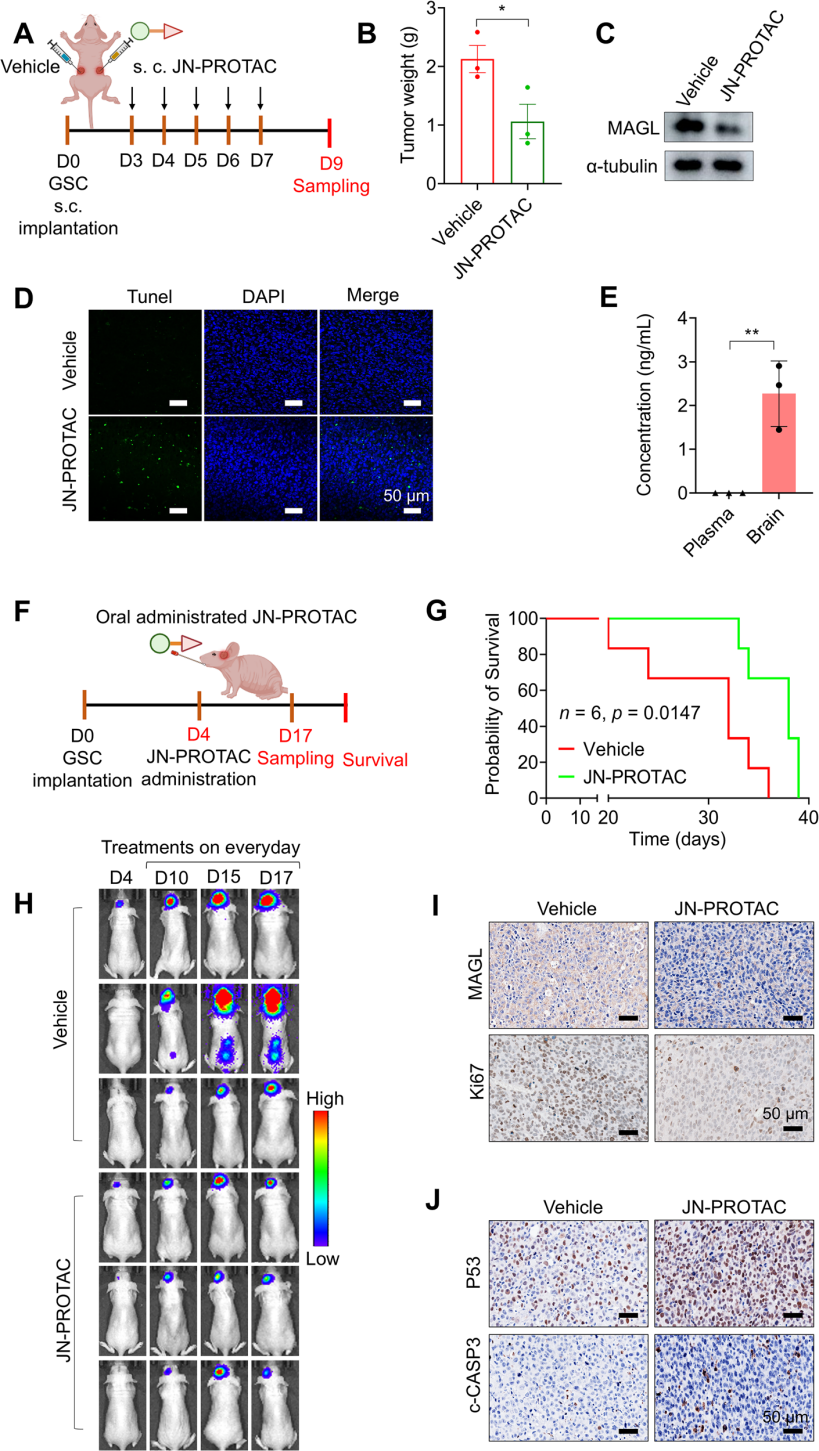
JN-PROTAC inhibits GBM growth in vivo and extends the survival rate of GSC-bearing mice. **A** The treatment strategy for the X01-Luciferase GSCs-bearing nude mice model involved subcutaneously injecting JN-PROTAC (10 mg/kg) directly at the tumor site, administered five times a week. **B** Quantification of the tumor weight derived from X01 GSCs (2×10^6^) subcutaneously implanted mice model. Mice were treated with JN-PROTAC (9 days, 10 mg/kg) or vehicle for 9 days. Data are presented as mean ± SD (*n* = 3, independent experiments), two-tailed Student’s t-test (*p = 0.047). **C** Immunoblotting for MAGL expression in tumor tissues collected from the subcutaneous mice model treated with JN-PROTAC or vehicle. **D** Representative Tunel staining immunofluorescence images of tumor tissues from subcutaneously implanted X01 GSC-bearing mice treated with JN-PROTAC or vehicle (9 days, 10 mg/kg). Green: Tunel signal, blue: DAPI staining for nucleus. Scale bar, 50 μm. **E** Mice were treated orally with JN-PROTAC at a dosage of 30 mg/kg, and serum and brain homogenate samples were collected for HPLC analysis at the indicated time points. Data are presented as mean ± SD (*n* = 3, independent experiments), two-tailed Student’s t-test (**p = 0.0063). **F** Strategy for treatment experiments in orthotopic X01-Luciferace GSCs tumors bearing nude mice model with orally administered JN-PROTAC. The mice received JN-PROTAC orally (30 mg/kg) five times per week for a consecutive period of 3 weeks (total 15 times). **G** Kaplan–Meier survival curves of mice implanted with 1 × 10^5^ X01-Luc GSCs and treated with JN-PROTAC or vehicle (*n* = 6 in each group). *p = 0.0147, log-rank test. **H** Representative luminescence images of X01-Luc GSCs bearing mice with JN-PROTAC or vehicle treatments. **I**, **J** Immunohistochemical (IHC) analysis of MAGL, Ki67 (**I**), P53 and cleaved-Caspase3 (c-CASP3) (**J**) in the orthotopic xenograft mouse model. Immunohistochemical scale bar, 50 µm. Samples are collected after 24 days post-implantation.

Further, we tested the in vivo anti-GBM potential of the JN-PROTAC in a GSCs subcutaneous implantation model in nude mice, where tumor growth and targeted protein degradation are easy to monitor (Fig. 4A). Following subcutaneous injection, the JN-PROTAC treated group exhibited a significant reduction in both tumor weight and tumor volume compared to the control group (Fig. 4B and Supplementary Fig. 10A, B). Additionally, MAGL protein expression was reduced in tumor lysates from the JN-PROTAC treated group (Fig. 4C) and the apoptosis Tunel signals were detected in the tumor region (Fig. 4D). Immunohistochemical analysis further demonstrated that, following PROTAC treatment, MAGL expression was reduced, while cleaved-Caspase3 and P53 expression levels were increased (Supplementary Fig. 10C–E), which suggested JN-PROTAC can act its functional targeted protein degradation role in vivo and trigger cell death of GSCs. Encouraged by the subcutaneous implantation model, we proceeded to assess its efficacy in the patient-derived xenograft (PDX) model, wherein X01 GSCs were implanted into the brain of nude mice (Fig. 4F). Given the scarcity of clinically available orally administered drugs for GBM, we opted to evaluate the in vivo antitumor potential via stomach lavage using syringe injection. The PDX model was established by implanting patient derived X01 GSCs into the nude mice according to our well-established model [32]. We first employed the high-performance liquid chromatography (HPLC) to assess the concentrations of JN-PROTAC in plasma at 1, 2, 3, 4, 5, 6, 7, and 8 h post injection, as well as in the accumulation in brain at 8 hours following a single administration of JN-PROTAC. The results showed that the plasma concentration reached a peak of 11 ng/mL at 2 h post-administration (Supplementary Fig. 11A), while the concentration in the brain at 8 h was approximately 2.1 ng/mL (Fig. 4E). These findings suggest that JN-PROTAC could partially penetrate the blood-brain barrier (BBB) in mice, making it possible for further evaluation of its in vivo anti-tumor effects. We then conducted an orthotopic xenograft mice model bearing X01-luciferase GSCs, at day 4 post-implantation, JN-PROTAC was orally administrated in a PEG-tween mixture daily for 15 times (Fig. 4F). Survival analysis of the JN-PROTAC group revealed a significant extension of the survival period compared to the vehicle injection group (Fig. 4G), indicating its potential as an anti-GBM agent via oral administration. Additionally, monitoring the luciferase signal of luciferase reporter-transfected X01 GSCs showed evident inhibition of tumor progression in the JN-PROTAC treatment groups (Fig. 4H), and there were no significant body weight changes in the JN-PROTAC-treated group throughout the treatment period. In contrast, the control group exhibited a noticeable decrease in body weight, likely due to the increasing tumor burden (Supplementary Fig. 11B). To further validate downstream molecular signaling responses, tumors were harvested on day 24 post-implantation and subjected to a series of histological staining assays. MAGL-targeted degradation was observed in the implanted tumor region, accompanied by reduced tumor viability as evidenced by decreased Ki67 expression, a classic tumor proliferative marker (Fig. 4I). Moreover, elevated levels of P53 and cleaved-Caspase3 in the orthotopic xenograft tumor region were detected (Fig. 4J), suggesting effective tumor inhibition. These findings collectively underscore the potential of JN-PROTAC as a MAGL PROTAC degrader to offer synergistic anti-GBM effects through simultaneous MAGL degradation and P53 activation, with demonstrated oral administration activity.

## Discussion

The clinical management of GBM is severely hampered by the limited therapeutics available, exacerbated by its hypermutation and highly invasive nature, leading to frequent drug resistance and posing significant challenges to patient outcomes. Recognizing the pivotal role of GSCs in GBM recurrence, infiltration, and resistance underscores the urgent need for GSC-targeted therapeutics, yet the exploration of such agents remains constrained. In our effort to augment the efficacy of GSCs treatment through MAGL targeting, as elucidated in our previous research, and to circumvent potential translational obstacles arising from the nonspecific effects of MAGL inhibitors, we proposed to enhance the specificity of MAGL targeting intervention by incorporating the ligand of the highly expressed MDM2 E3 ligase in GBM into the design of the PROTAC. Through a cross-disciplinary approach, this study pioneers the development of the first orally administrable GBM PROTAC tailored for GSCs, representing an advancement in GBM treatment strategies. Notably, this PROTAC may confer enhanced activity in various tumor cell types expressing MAGL and high levels of MDM2, potentially mitigating side effects in normal cells with lower expression levels. Proteomics analysis unequivocally identified MAGL as the primary target upon PROTAC treatment. Functional annotation of DEGs revealed that lipid metabolism signaling, the principal pathway through which MAGL exerts its regulatory function, is prominently enriched, further substantiating the effect of the PROTAC. In contrast, while JZL184 also induces time- and dose-dependent elimination of MAGL, its selectivity and efficacy in MAGL modulation are markedly inferior to that of the PROTAC. Notably, proteomics profiling disclosed that JZL184 exerts a significant impact on a broader spectrum of signaling cascades beyond lipid metabolism, thereby suggesting the presence of potential off-target effects. Given the ubiquitin-proteasome axis mediated by PROTAC, even at suboptimal dosage or following drug clearance, the compound can persistently elicit sustained therapeutic effects via this functional loop. This enduring action is contrasts to parental inhibitor, which are incapable of maintaining their efficacy once eliminated from the system, thereby underscoring the unique and prolonged pharmacodynamic advantage of PROTAC-based therapies. This distinction accentuates the superior specificity and sustained action of the PROTAC, further highlighting its translational potential for clinical application. Although the current iteration of the PROTAC demonstrates substantial efficacy, we acknowledge that there remains room for improvement, particularly in terms of selectivity. The optimization of JN-PROTAC for clinical translation will require comprehensive improvements to both the MAGL-specific binding derivatives and the linker. Enhancing selectivity will involve refining the linker structure and conducting systematic screening of alternative E3 ligases, along with warhead binding affinity analyses, to ensure more precise targeting of MAGL. Developing a library for screening and performing these analyses will be crucial for the hit-to-lead process, optimizing both binding affinity and therapeutic index. The current administration route for the designed PROTAC is oral, although there is no specific design for enhancing blood-brain barrier (BBB) penetration. In this study, the survival of the experimental mice is prolonged, potentially due to the glioma partially compromising the integrity of the BBB. As a proof-of-concept study, the JN-PROTAC could partially penetrate the BBB (Fig. 4E) and function in targeted protein degradation and tumor suppression in vivo. Future applications may leverage nanodelivery strategies to improve circulation and tumor targeting performance, potentially achieving better outcomes for translational potential.

## Methods

### Cell culture and chemical synthesis

The murine melanoma cells (B16F10) and the human breast cancer cells (MDA-MB-231) were maintained in Dulbecco’s modified Eagle’s medium (DMEM) supplemented with 10% fetal bovine serum (HyClone), 1% non-essential amino acids (Oricell), and 1% penicillin/streptomycin (P/S; HyClone). Patient-derived GBM stem cells (X01, 528) were cultured in DMEM/F-12 (HyClone) mixed with B27 (Invitrogen), EGF (10 ng/mL, R&D Systems), bFGF (10 ng/mL, R&D Systems), and 1% penicillin/streptomycin (P/S; HyClone). All cells were repeatedly screened for mycoplasma and then cultured at 37 °C in an incubator containing 5% CO_2_. MG-132 (Cat. No. T2154), and TAK-243(Cat. No. T16974) were purchased from Shanghai Topscience Co., Ltd., and JZL184 (Cat. No. 3836) was received from Tocris Bioscience. For detailed chemical synthesis and characterization please see Supplementary Information.

### Bioinformatics analysis E3 ligase screening and molecular docking

Basically, the general E3 ligase candidates expression matrix was collected from TCGA microarray dataset and RNA-seq dataset from UCSC xena portal (https://xena.ucsc.edu/public), CPTAC RNA-seq and proteomics dataset from the CPTAC portal (https://cptac-data-portal.georgetown.edu/), various E3 ligase candidates expression in GBM were compared to Normal tissue by *p*-value and fold change for the optimal candidate. The molecular docking is used to analyze the binding properties between different compounds and the aimed protein. The 3D structure file of MAGL protein (PDB ID: 3HJU) was downloaded from the Protein Structure Database (https://www.rcsb.org). Then we used the Gaussian 16 program package [33] for quantum mechanical optimization of JZL184 (Cat. No: 1101854-58-3), JZL184 analog and conjugated PROTACs. The geometry optimization was carried out at B3LYP-D3 level of theory with the 6-31 G(d) basis set. According to the location of the original ligand, we set the docking range to be near the active site of the MAGL, with the inner box size of 10 × 10 × 10 Å and the outer box size of 45 × 45 × 45 Å. In order to improve the accuracy and efficiency of the docking, we set the exhaustiveness parameter to be 12, and the energy range parameter to be 4. Next, the intermolecular affinity energies were calculated using AutoDock Vina software [34]. Data showed the affinity energies of -10.6 kcal/mol, -9.6 kcal/mol, and -7.2 kcal/mol for JZL184, JZL184 analog, and the JN-PROTAC, respectively. LigPlot+ software was used to analyze the molecular interaction residues and types of forces [35] and PyMOL software V2.5 (http://www.pymol.org/) was used to visualize the docked conformations and to map the molecular interactions.

### Cell treatment

For cellular experiments, both JN-PROTAC and JZL184 were freshly dissolved in DMSO before utilization. Briefly, the X01 and 528 GSCs (5 × 10^5^ cells) were subjected to treatment with JZL184 at different doses, and DMSO solvent was used as the negative control. After 24 h of incubation, the cells were harvested for immunoblotting. Moreover, GSCs were subjected to JN-PROTAC treatment at a concentration of 500 nM for incubation periods of 1, 2, 4, 8, 12, and 24 hours before harvesting. The B16F10, MDA-MB-231, X01, and 528 GSCs were exposed to JN-PROTAC at different doses, while DMSO served as the negative control. After 24 h of incubation, the cells were harvested for immunoblotting. The GSCs were harvested for immunoblotting after 24 h by treating with JN-PROTAC, TAK-243, or MG-132, and combination treatments with JN-PROTAC and TAK-243 or MG-132, respectively.

### Antibodies and immunoblotting

Following treatment under various conditions, the extracted cells were washed once with ice-cold 0.9% NaCl solution and centrifuged at an appropriate speed of 8000 rpm for 5 min at 4 °C. Next, the cells were lysed with RIPA buffer containing protease inhibitors (Roche) and then incubated on ice for 30 min to ensure complete lysis. The protein concentration in the supernatants was assessed using the BCA Protein Quantification Kit (Cat. No. B2290CA, Vazyme). The protein was normalized, and the samples were reduced in 5×Omni-Easy™Protein Sample Loading Buffer (Cat. No. LT101, YAMEI) and denatured at 100 °C. Proteins were first separated using precast 7.5–12.5% Tris-glycine gels made by PAGE Gel Fast Preparation Kit (Cat. No. PG110, Epizyme Biotech), and then transferred to 0.22-µm pore size polyvinylidene difluoride (PVDF) membranes (Millipore) under the condition of 250 mA constant current for 2 h, followed by blocking with 5% skim milk (BD) for 1 h at room temperature. Next, the membranes were incubated with primary antibodies against MAGL (1:1000, Sangong biotech, Shanghai), MDM2 (1:1000, Cell Signaling Technology; 1:1000, Proteintech), PARP (1:1000, Cell Signaling Technology), cleaved-PARP (1:1000, Cell Signaling Technology), Caspase3 (1:1000, Cell Signaling Technology), cleaved-Caspase3 (1:1000, Cell Signaling Technology), α-tubulin (1:1000, Proteintech) overnight at 4 °C. The membranes were washed three times in 1% TBST and incubated with horse radish peroxidase (HRP)-conjugated secondary antibodies for 1 h at room temperature. The immunoreactive bands were visualized using the Amersham ECL Prime Western Blotting detection reagent (GE Healthcare).

### Cell proliferation assays

The X01 GSCs were plated at a density of 500 cells/ well in 96-well plates (80 μL/well) containing DMEM/F-12 medium supplemented with B27, epidermal growth factor (EGF; 10 ng/mL), and basic fibroblast growth factor (bFGF; 5 ng/mL). After 24 h, 20 µL of culture medium containing JN-PROTAC at different concentrations was added to each well. The outer well of the 96-well plate were filled with 100 μL of PBS to avoid evaporation of medium from the treatment wells. Following treatment for 0 days, 2 days, 4 days, and 6 days, the cell viability was measured with Cell Counting Kit-8 (CCk8) by a SpectraMax^®^ i3x Microplate Reader (Molecular Devices) according to the manufacturer’s protocol. The result was analyzed using GraphPad Prism.

### Sphere formation assay

The X01 and 528 GSCs were plated at a density of 100 cells/plate in 12 well plates containing DMEM/F-12 medium supplemented with B27, EGF, and bFGF. Following 24 h culture, cells were subjected to treatment with 0.5 μM JZL184 or 0.5 μM JN-PROTAC, while DMSO served as the negative control. Then, the cell culture samples were incubated in a humidified atmosphere under 5% CO_2_ at 37 °C for 14 days. Later, the plates were examined for sphere formation using an inverted microscope. The spheres with diameter >50 μm were then counted.

### Flow cytometry analysis

The X01 GSCs were treated with different concentrations of JN-PROTAC for 24 h, while untreated cells served as a control group. Following cell collection and NaCl washing, Annexin V-FITC Apoptosis Detection Kit (Cat. No. C1062S, Beyotime) staining was performed in accordance with the manufacturer’s instructions. Utilizing the BD LSRFortessa, data were collected and analyzed using the FACS Diva and FlowJo software.

### Mouse models

All animal experiments are following standard protocols approved by the Henan University Laboratory Animal Center and Ethics Committee (approval number HUSOM2022-450). Female BALB/c nude mice aged 5 weeks were purchased from SPF (Beijing) BIOTECHNOLOGY Co., Ltd., and housed under specific pathogen-free conditions. Before conducting experiments, each animal was assigned at random by body weight. For the orthotopic mouse model, X01-luc GSCs were first suspended in a culture medium and then transplanted into the left striatum of mice via stereotactic injection (1 × 10^5^ cells/mouse). The injection was administered at the coordinates of 2.2 mm lateral to the midline, 0.2 mm posterior to the bregma, and at a depth of 3.5 mm. The animals were randomly assigned into two groups and orally administered with (1) vehicle control, or (2) JN-PROTAC 30 mg per kg body weight, five times a week. JN-PROTAC for oral administration was formulated in DMSO (1%), polyethylene glycol (PEG) 300 (45%), and PBS (54%). The body weight and tumor size (observed by luminescence intensity) of the mice were monitored and recorded. For the subcutaneous mouse model, X01-luc cells (2 × 10^6^ cells) were first suspended in 150 μL of DMEM/F-12 medium and then were subcutaneously (s.c.) implanted in the right and left armpit of BALB/c nude mice, tumor growth was monitored daily, and tumor size was measured using digital calipers. Treatment started once the tumor diameter reached 0.5 cm, the animals were randomly assigned into two groups (Vehicle and JN-PROTAC group). JN-PROTAC for in situ administration was formulated in DMSO, and JN-PROTAC was administered via in situ injection at 10 mg/kg body weight in a 50 μL vehicle, five times a week. Control mice received 50 μL vehicle via s.c. injection. In addition, mice were euthanized when their maximum tumor size reached the humane endpoint in the subcutaneous implanted mice model, defined according to institutional policy concerning tumor endpoints in rodents. When a 20% reduction in weight was achieved in the orthotopic xenograft mice model, the mice were euthanized. Subsequently, each various mice tissues were collected and immersed in 4% paraformaldehyde for 24 h at 4 °C for further analysis. GraphPad PRISM software was used to analyze their survivability.

### Protein extraction from tumors for immunoblotting

The tumor samples from the subcutaneous mouse model were immediately frozen at -80 °C for later use once isolated and washed. To lyse the tumor tissue, 1 mL of PMSF-supplemented RIPA lysis buffer was used. The tumor tissues were homogenized using a hand homogenizer while being kept on ice. Next, the samples were then incubated for 2 h on ice for complete lysis. Subsequent sample preparation and immunoblotting were conducted using the methodology outlined in the immunoblotting procedure.

### Immunohistochemistry (IHC) and immunofluorescence staining

Immunohistochemical (IHC) staining of ki67 (1:500, R&D Systems) was conducted using the SABC immunohistochemical staining kit (Cat. No. SA1021, Boster) following the manufacturer’s instructions. Endogenous peroxidase activity was eliminated by incubating sections with 3% hydrogen peroxide for 5 min, followed by an antigen retrieval process using citrate buffer (0.01 M). The sections were then blocked with 5% BSA for 30 min at 37 °C in dark conditions. After diluting the primary antibody with diluent buffer, the sections were incubated at 4 °C in a humidified chamber for an overnight period. On the second day, the sections underwent incubation with horse radish peroxidase (HRP)-conjugated secondary antibodies for 30 min as above. Then the sections were subsequently incubated at 37 °C for an additional 30 min with SABC. The tissue sections were then stained with 3,3′-diaminobenzidine (DAB, AR1020) as the chromogen. Finally, the sections were counterstained with hematoxylin, dehydrated, dried, and affixed with an organic mounting medium. Tunel fluorescence staining was performed following standard protocols, where fresh tissue sections were incubated with an anti-Tunel antibody (1:500, Abcam) overnight at 4 °C. Then secondary fluorochrome-conjugated antibodies (Alexa 568, 1:500, Thermofisher Scientific) were incubated, followed by nuclei staining with DAPI (1:5000; Sigma) staining for 5 min. Then the fluorescence images were captured using a Zeiss LSM 880 confocal laser scanning microscope (Carl Zeiss, Thornwood, NY, USA).

### High-Performance Liquid Chromatography (HPLC)

HPLC was used to analyze JN-PROTAC concentration in plasma and brain tissue. 6 weeks old nude mice were treated with JN-PROTAC at 30 mg/kg, followed by blood collection at 1, 2, 3, 4, 5, 6, 7, and 8 h, and brain collection at 8 h. Plasma samples were mixed with acetonitrile and allowed to extract at 4 °C overnight. Brain tissue samples were homogenized in acetonitrile and similarly incubated overnight at 4 °C. The following day, samples were centrifuged at 4 °C for 10 minutes, and the supernatant was collected for HPLC analysis. The analysis was conducted using an Agilent InfinityLab Poroshell 120 Chiral-V (4.6 mm x 100 mm, 2.7 µm) with a UV-Vis detector set to 280 nm. A gradient elution was performed using solvent A (water with 0.1% TFA) and solvent B (acetonitrile with 0.1% TFA), starting at 10% B and increasing to 90% over 10 minutes, followed by re-equilibration to the initial conditions. For each analysis, 10 µL of the prepared sample was injected into the HPLC system.

### Co-immunoprecipitation

Co-immunoprecipitation (co-IP) was performed using Invitrogen Dynabeads Protein A/G Immunoprecipitation Kit (Thermo Fisher Scientific) and anti-MDM2 antibody (proteintech). Briefly, X01 and 528 GSCs were treated with DMSO or JN-PROTAC (10 μM) for 48 h before harvest. The cells were washed twice with ice-cold PBS and then IP lysis buffer (1 mL) in the presence of 1% protease inhibitor was used to extract protein. Subsequently, magnetic beads (30 µL/sample) were separated from the solution using a magnetic strand. 100 µL of protein lysate was used as input and the remaining cell lysates were incubated with Protein A/G Dynabeads preincubated with the indicated antibody or with anti-MDM2 antibody at 4 °C overnight with rotation. The magnetic beads were then washed 3-4 times with IP buffer containing 1% protease inhibitor and 20% NaCl (1 M). Immunoprecipitates and input were boiled at 100 °C for 10 min in a protein loading buffer (EpiZyme) for further immunoblotting analysis.

### Proteomics sequencing and Functional analysis

Proteins were extracted from X01 GSCs treated with DMSO vehicle, JZL184 and JN-PROTAC (three biological replicates per group) using DB lysis buffer (8 M urea, 100 mM TEAB, pH 8.5) and processed with ultrasonication, reduction (1 M DTT, 56 °C, 1 h), alkylation (iodoacetamide, room temperature, 1 h, dark), and acetone precipitation (-20 °C, 2 h). The pellet was washed, air-dried, and dissolved in DB buffer. LC-MS/MS was performed on a ThermoFisher Vanquish™ Neo UHPLC coupled with an Orbitrap Astral mass spectrometer in DIA mode. Raw data were analyzed using DIA-NN software for both protein identification and quantification, with searches conducted against the UniProt database. Differentially expressed proteins (DEPs) were identified based on fold change, calculated as the ratio of average values between groups. Gene Ontology (GO) was used for functional annotation of DEPs.

### Statistics

All data are presented as means ± SD from three independent experiments. Survival curves were plotted using the Kaplan–Meier method. Multiple datasets were compared using ANOVA with the log-rank test, while two-dataset experiments were compared using a two-tailed Student’s t-test. *p*-values < 0.05 were determined as statistically significant. Each experiment was performed in triplicates.

## Supplementary information

Supplemental information-MAGL PROTAC

Western blot gels raw data

## Data Availability

The raw proteomics profiling data generated in this study has been uploaded in iProtX database (https://www.iprox.cn/page/home.html, Project ID: IPX0010392000). Additional data generated in this study are available upon request from the corresponding author.
