# Supplementary material for: MAGL targeted PROTAC degrader simultaneously enhances P53 for synergistic treatment of glioblastoma stem cell
Source: Cell Death Discov. 2025 Mar 20;11:109. doi: 10.1038/s41420-025-02392-1 (PMC11926070; doi:10.1038/s41420-025-02392-1)
Supplement: Supplementary file 1 — Supplemental information-MAGL PROTAC [file 41420_2025_2392_MOESM1_ESM.pdf]

Supplemental information

MAGL Targeted PROTAC degrader Simultaneously Enhances P53 for Synergistic Treatment of Glioblastoma Stem Cell

Zheng Yuan,[1,2,5] Meixia Guo,[1,2,5] Yue Zhang,[1,2,5] Yilin Deng,[1,2,3,5] Biao Sun,[1,2] Yaning Hou,[1,2] Xin Wang,[1,2] Xiong Jin,[1,2] Yang Liu,[1,4,✉] Bingyang Shi,[1,2,✉] Jinlong Yin [1,2,4,✉]

✉email: jlyin@henu.edu.cn; liuyang.jcbi@henu.edu.cn; bingyang.shi@mq.edu.au

This file including:

Methods of chemical synthesis
Supplementary Fig. 1-11

Experimental Procedures

Chemical synthesis

The synthetic schemes and chemical structures of JN-PROTAC are presented in Supplementary figure 1 Detailed synthetic procedures are provided below. Oven-dried glassware was used for experiments involving moisture and/or air-sensitive components. Analytical thin-layer chromatography (TLC) was used to monitor all reaction processes. Merck 60 F254 precoated silica gel plates (0.2 mm thickness) were employed and visualized under UV radiation. The products were purified using silica gel packed columns and equilibrated with petroleum ether. Proton nuclear magnetic resonance (^{1}H NMR) spectroscopy was performed using Bruker Advance III NMR spectrometer to analyze the chemical structures.

Synthesis of JZL184 analog

Synthesis of N-Boc-Piperidine-4-carboxylic acid methyl ester **(3)**

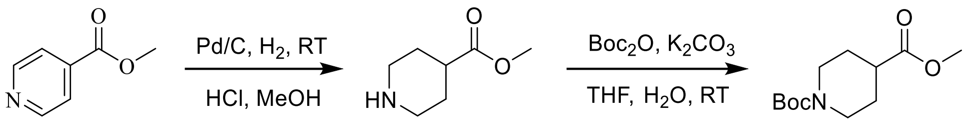

1 **2** **3**

From methyl isonicotinate **1**, methyl 4-piperidinecarboxylate **2** was generated by acidifying a methanol solution with HCl and stirring for 24 h at 25 °C under N_2 atmosphere while using Pd/C as the catalyst. Then, methyl 4-piperidinecarboxylate **2** (14.32 g, 79.7 mmol), di-tert-butyl dicarbonate (Boc_2O; 21.82 g, 10 mmol), K_2CO_3 (13.82 g, 10 mmol) were reacted in the mixture of THF (50 mL) and H_2O (50 mL) at room temperature for another 24 h. The crude product was obtained by evaporation of the solvent under reduced pressure and then purified by silica gel column chromatography using petroleum ether and ethyl acetate (10:1) as eluents to give the compound N-Boc-piperidine-4-carboxylic acid methyl ester **3**.

Synthesis of compound **(6)**

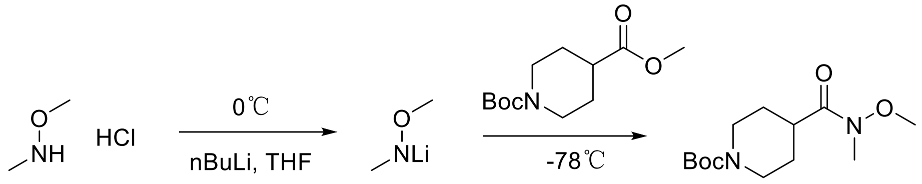

4 **5** **6**

N, O-dimethylhydroxylamine. HCl **4** (97.5 g, 100 mmol) was dissolved in 100 mL THF. Next, nBuLi (100 mL, 160 mmol) was added and the reaction was stirred for 30 minutes at 0 °C, followed by the addition of N-Boc-piperidine-4-carboxylic acid methyl ester **3** (11.1 g, 40 mmol). The reaction was stirred for 2 h at -78 °C. The crude product was prepared by evaporation of the solvent in reduced pressure and then isolated by silica gel column chromatography using petroleum ether and ethyl acetate (1:1) as eluents to produce compound **6**.

Synthesis of compound **(9)**

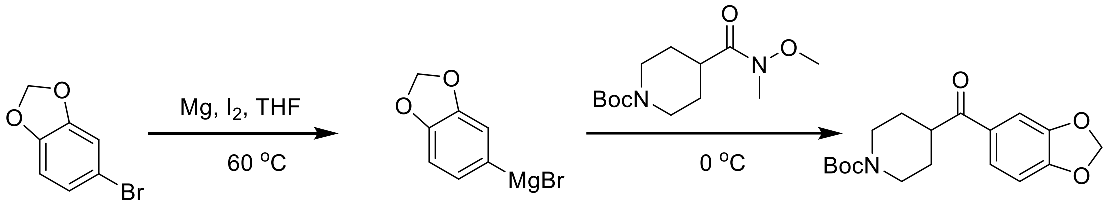

7 **8** **9**

4-bromo-1,2-(methylenedioxy) benzene **7** (12.3 g, 61.2 mmol) was dissolved in dry THF, and refluxed with Mg (1.8 g, 75 mmol) and I_2 (0.01 g) under nitrogen atmosphere at 60 °C for 1 h. Compound **6**, dissolved in THF (9.4 g, 30.6 mmol), was subsequently introduced into the reaction system at 0 °C. The reaction system was reacted for 2 h. To extract the mixture, ethyl acetate was used and the resulting organic phases were washed with NaCl, dried over Na_2SO_4, filtered, and evaporated under reduced pressure. The compound **9** was obtained by purifying the crude product through silica gel column chromatography using petroleum ether and ethyl acetate (3:1) as eluents.

Synthesis of compound **(12)**

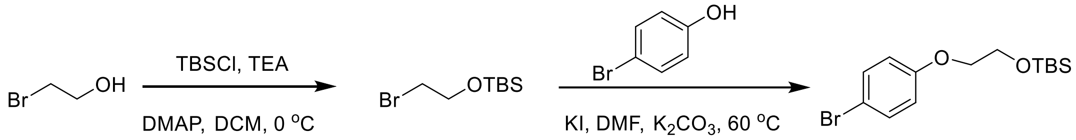

10 **11** **12**

Under a nitrogen atmosphere, 2-bromoethanol **10** (12.5 g, 100 mmol) and TEA (20.24 g, 200 mmol) were dissolved in DCM (100 mL), and the temperature of the reaction system was lowered to 0 °C. 10.x g portions of tert-butyldimethylsilyl chloride (TBSCl), equivalent to 18.08 g (120 mmol), were introduced in parts of 1.8 g at a time. Finally, 0.12 g of 4-dimethylaminopyridine (DMAP; 1 mmol) was added, the temperature was gradually raised to 25 °C, and the reaction was stirred for 8 h to obtain compound **11**. Subsequently, the resulted compound **11** (23.92 g, 100 mmol) was reacted with 4-bromophenol (17.30 g, 100 mmol) under K_2CO_3 (13.82 g, 200 mmol), and KI (1.66 g, 10 mmol) catalytic system in DMF (100 mL) at 60 °C (3 h, N_2 atmosphere). The compound **12** was obtained by purifying the crude product through silica gel column chromatography using petroleum ether and ethyl acetate (100:1) as eluents, after the mixture was extracted with ethyl acetate and the combined organic phases were washed with saline, dried over Na_2SO_4, filtered, and evaporated to dryness under reduced pressure.

Synthesis of compound **(14)**

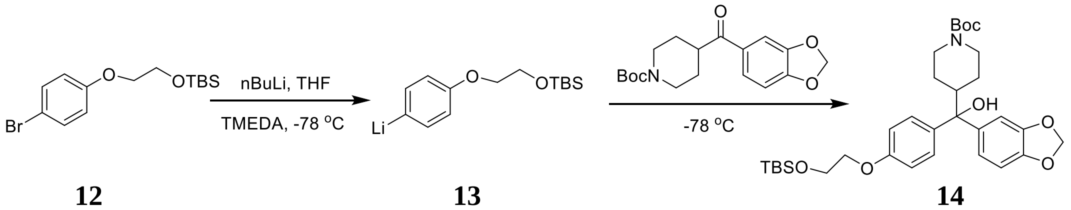

12 **13** **14**

Compound **12** (15.72 g, 47.5 mmol) and N,N,N',N'-tetramethylethylenediamine (3.86 g, 33.21 mmol) were mixed in the 100 mL dry THF under nitrogen atmosphere. The reaction system was cooled down to -78 °C, then nBuLi solution (20.8 mL, 33.21 mmol) was slowly added and the reaction was stirred under the same condition for 30 min. Subsequently, compound **9** (8.72 g, 23.21 mmol) was dissolved in 50 mL of THF and added to the reaction

mixture. The resulting mixture was then stirred at a temperature of -78 °C for a duration of 30 minutes. Ethyl acetate was used to extract the mixture. The combined organic phases were washed with NaCl, dried on Na_2SO_4, filtered and evaporated to dryness under reduced pressure. The crude product was purified by silica gel column chromatography using petroleum ether and ethyl acetate (3:1) as eluents to yield compound **14**.

Synthesis of JLZ184 analog **(17)**

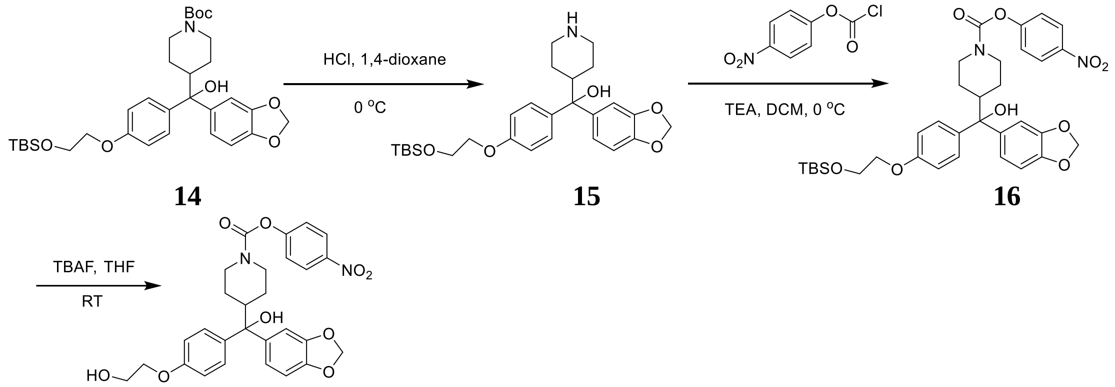

14 **15** **16**

JLZ184 analog (17)

In the absence of O_2, compound **14** (4.9 g, 7.9 mmol) was dissolved in 1,4-dioxane (10 mL), and then 4 M HCl was drop-wisely added to the above reaction system at 0 °C. After that, the reaction was slowly warmed up to room temperature and stirred for 1 h to deprotect Boc and obtain the product compound **15**. Next, add compound **15** (3.8 g, 7.9 mmol) and TEA (1.6 g, 15.8 mmol) to 10 mL of DCM. The temperature of the reaction system was dropped to 0 °C and gradually introduced 4-nitrophenyl chloroformate (2.4 g, 11.9 mmol). The reaction system was gradually heated to reach 25 °C and thereafter continued reaction for 1 h to get the result compound **16**. Compound **16** (5.2 g, 8 mmol) and tetra-n-butylammonium fluoride (TBAF; 5 g, 15.9 mmol) were stirred in THF (8 mL) and reacted at room temperature for 30 min to deprotect the hydroxyl group. The mixture was extracted with ethyl acetate. The combined organic phases were washed with brine, dried over Na_2SO_4, filtered, and evaporated to dryness under reduced pressure. The crude product was purified by silica gel column chromatography using petroleum ether and ethyl acetate (1:1) as eluents to afford the product JLZ184 analog (**17**). The chemical structure of the JZL184 analog was characterized by ^{1}H-NMR spectroscopy.

^{1}H NMR (300 MHz, CDCl3) δ 8.19 (d, J = 9.1 Hz, 2H), 7.34 (d, J = 8.8 Hz, 2H), 7.27 - 7.18 (m, J = 9.1 Hz, 2H), 6.87 (dd, J = 19.4, 8.0 Hz, 4H), 6.73 (d, J = 8.6 Hz, 1H), 5.90 (s, 2H), 4.27 (t, J = 11.5 Hz, 2H), 3.99 (dd, J = 21.1, 16.5 Hz, 4H), 2.93 (dt, J = 39.6, 12.2 Hz, 2H), 2.48 (q, J = 11.4 Hz, 3H), 1.72 - 1.30 (m, 4H).

Synthesis of Nutlin-3 analog

Synthesis of compound (**20**)

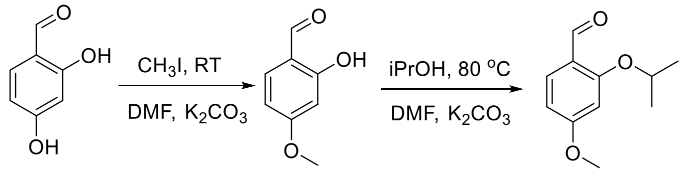

18 **19** **20**

Under N2 conditions, 2,4-dihydroxybenzaldehyde **18** (13 g, 100 mmol), K_2CO_3 (16.6 g, 120 mmol), and CH_3I

(14.19 g, 100 mmol) were dissolved in DMF (80 mL), and the reaction was stirred for 12 h at room temperature to obtain the product compound **19**. Then compound **19** (15.3 g, 100 mmol), K_2CO_3 (27.64 g, 200 mmol), iPrOH (20.4 g, 120 mmol) were dissolved in DMF (75 mL) and stirred at reflux condition (80 °C) for 3 h. The mixture was extracted with ethyl acetate. The combined organic phases were washed with NaCl, dried over Na_2SO_4, filtered, and evaporated to dryness under reduced pressure. The crude product was purified by silica gel column chromatography using petroleum ether and ethyl acetate (8:1) as eluents to obtain the compound **20**.

Synthesis of compound (**23**)

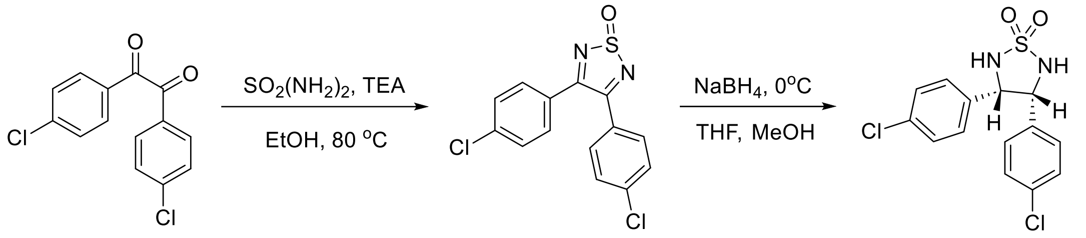

21 **22** **23**

1,2-bis-(4-chlorophenyl)-1,2-ethanedione **21** (55.82 g, 200 mmol), sulfanilamide (28.81 g, 200 mmol), $SO_2(NH_2)_2$ (28.81 g, 300 mmol), and TEA (8.36 mL, 60 mmol) were dissolved in absolute ethanol (1200 mL), refluxed at elevated temperature for 18 h. Next, the extra solvent was evaporated (600 mL), and the reaction was continued refluxing for 22 h to obtain the compound **22**. At 0 °C, compound **22** (9.3 g, 21.8 mmol) was dissolved in a mixture of MeOH and THF (1:1, 20 mL), and subsequently added $NaBH_4$ (6.24 g, 165 mmol). Stirring the reaction mixture at 0 °C for 1 h. The mixture was extracted with ethyl acetate. The combined organic phases were washed with NaCl, dried over Na_2SO_4, filtered, evaporated to dryness under reduced pressure, and yielded compound **23**.

*Synthesis of compound (**25**)*

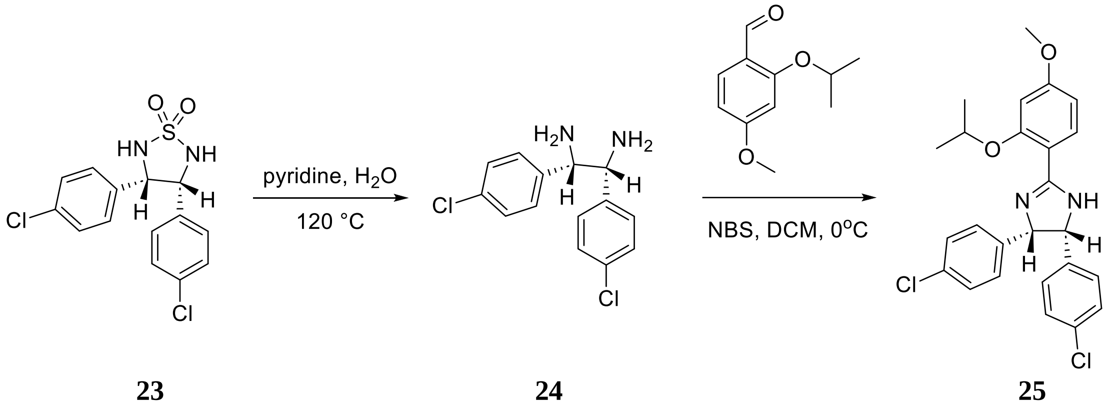

23 **24** **25**

In the next step, compound **23** (9.4 g, 27.5 mmol) was re-dissolved in a mixture solution containing pyridine (15 mL) and water (1.5 mL). The reaction system was subjected to reflux stirring for 18 h at 130 °C that finally resulted into compound **24**. Following that, compound **24** (9.0 g, 3.2 mmol) and compound **20** (5.6 g, 28.8 mmol) were dissolved in DCM and the reaction mixture was stirred at 0 °C for 30 min. Then, N-bromosuccinimide (7.9 g, 44.5 mmol) was added. The reaction mixture was gradually warmed to room temperature and stirred for 12 h. The mixture was extracted with DCM. The combined organic phases were washed with NaCl, dried over Na_2SO_4,

filtered, evaporated to dryness under reduced pressure, and yielded compound **25**.

Synthesis of Nutlin-3 analog **(28)**

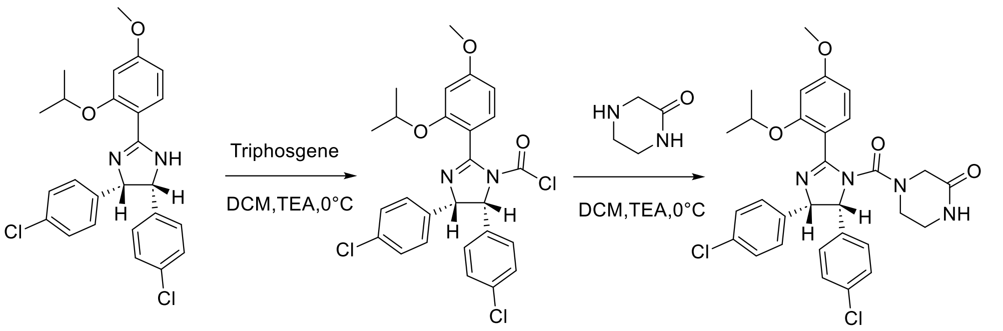

25 **26** **27**

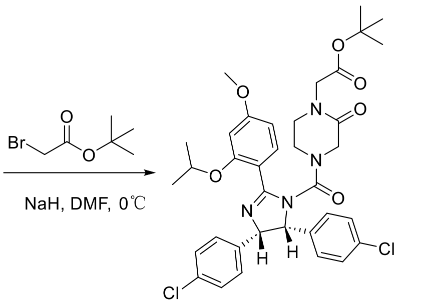

Nutlin-3 analog (28)

The compound **25** (2.28 g, 5 mmol) dissolved in 10 mL of DCM was combined with TEA (5.06 g, 5.5 mmol), and the reaction system was cooled down to 0 °C. Subsequently, triphosgene (4.45 g, 15 mmol) diluted in 10 mL of anhydrous DCM was drop-wisely added and stirred the reaction for 30 min to obtain the compound **26**. Later on, the intermediate **26** was further reacted with piperazin-2-one (0.5 g, 5 mmol) in the presence of TEA (2.53 g, 25 mmol) in DCM (20 mL). The reaction temperature was slowly raised to 25 °C and stirred the reaction for 1 h. The mixture was extracted with DCM. The crude product was purified by silica gel column chromatography using DCM and MeOH (20:1) as eluents to obtain the compound **27**. For the synthesis of Nutlin-3 analog (**28**), first the NaH (0.3 g, 7.5 mmol) and DMF (10 mL) are mixed under a N_2 environment at 0 °C with full stirring. Then, the solution of compound **27** (2.91 g, 5 mmol) dissolved in 5 mL DMF is added drop-wisely. The temperature is then gradually increased to ambient temperature and the reaction is stirred for 30 min. Upon the reaction completion, the generation of gas bubble ceased. Next, at 0 °C, tert-butyl bromoacetate (1.17 g, 6 mmol) was supplemented, and the reaction was further continued for 1 h. The mixture was extracted with ethyl acetate. The combined organic phases were washed with NaCl, dried over Na_2SO_4, filtered, and evaporated to dryness under reduced pressure. The crude product was purified by silica gel column chromatography using ethyl acetate, petroleum ether and MeOH (3:6:1) as eluents to obtain the resulted Nutlin-3 analog (**28**). The chemical structure of the Nutlin-3 analog was characterized by ^{1}H-NMR spectroscopy.

^{1}H NMR (300 MHz, CDCl3): δ 7.59 (d, J = 8.5 Hz, 1H), 7.26 (s, 1H), 7.06 (dd, J = 15.3, 8.4 Hz, 4H), 6.91 (dd, J = 21.2, 8.4 Hz, 4H), 6.55 (dd, J = 8.5, 2.2 Hz, 1H), 6.48 (dt, J = 2.1 Hz, 1H), 5.52 (dd, J = 26.9, 9.7 Hz, 2H), 4.62 (dt, J = 12.1, 6H), 4.62 (dt, J = 12.1, 6H) , J = 2.1 Hz, 1H), 5.52 (dd, J = 26.9, 9.7 Hz, 2H), 4.62 (dt, J = 12.1, 6.0 Hz, 1H), 3.98 (d, J = 17.3 Hz, 1H), 3.85 (s, J = 5.3 Hz, 3H), 3.82 - 3.64 (m, 3H), 3.55 - 3.26 (s, J = 3.3 Hz, 3H), 3.55 - 3.26 (m, 3H) 3.55 - 3.26 (m, 2H), 3.06 (t, J = 5.3 Hz, 2H), 1.43 (s, 9H), 1.37 (dd, J = 9.9, 6.0 Hz, 6H).

Synthesis of JN-PROTAC

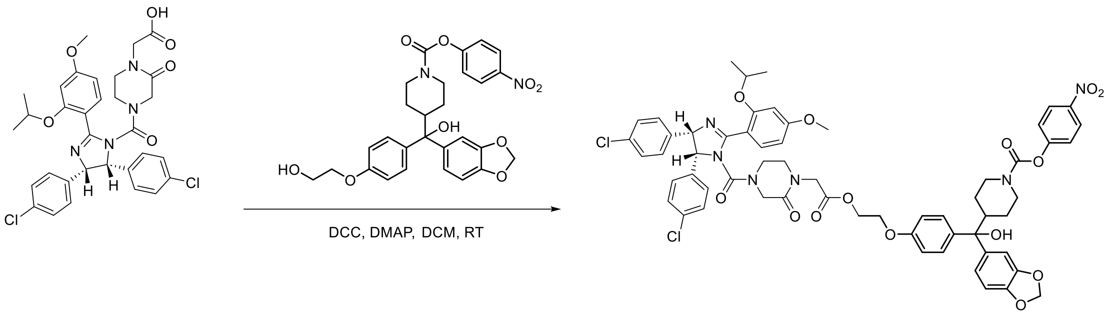

29 JZL184 analog JN-PROTAC

After the deprotection of Nutlin-3 analog (**28**) with TFA, the resulted compound **29** (0.184 g, 0.288 mmol), was esterified with JLZ184 analog (0.155 g, 0.288 mmol), under 4-dimethylaminopyridine (DMAP; 0.0035 g, 0.0288 mmol), and dicyclohexylcarbodiimide (DCC; 0.891 g, 0.432 mmol) catalytic system in DCM (1 mL) in the presence of nitrogen atmosphere for 1 h. The mixture was extracted with ethyl acetate. The combined organic phases were washed with brine, dried over Na_2SO_4, filtered, and evaporated to dryness under reduced pressure. The crude product was purified by silica gel column chromatography using petroleum ether and ethyl acetate (1:5) as eluents to obtain the product JN-PROTAC. The chemical structure of the final JN-PROTAC was characterized by ^{1}H-NMR spectroscopy.

^{1}H NMR (300 MHz, CDCl3): δ 8.23 (d, J = 9.1 Hz, 2H), 7.58 (d, J = 9.7 Hz, 1H), 7.42 - 7.22 (m, 5H), 7.06 (dd, J = 14.9, 8.4 Hz, 4H), 6.98 - 6.79 (m, 8H), 6.74 (d, J = 8.6 Hz, 1H), 6.60 - 6.44 (m, J = 20.8 Hz, 2H), 5.92 (s, 2H), 5.61 - 5.42 (m, J = 38.6 Hz, 2H), 4.61 (d, J = 24.1 Hz , 1H), 4.50 - 4.38 (m, 2H), 4.37 - 4.21 (m, J = 14.3 Hz, 2H), 4.19 - 4.03 (m, 3H), 3.93 - 3.80 (m, J = 18.7 Hz , 4H), 3.80 - 3.64 (m, J = 15.9 Hz, 2H), 3.54 - 3.27 (m, J = 13.2, 9.7 Hz, 2H), 3.12 - 2.81 (m, J = 62.2 Hz, 4H), 2.51 (t, J = 11.5 Hz, 1H), 2.27 (s, 1H), 1.62 (d, J = 11.5 Hz, 3H), 1.36 (d, J = 17.0 Hz, 9H), 0.96 - 0.77 (m, 1H).

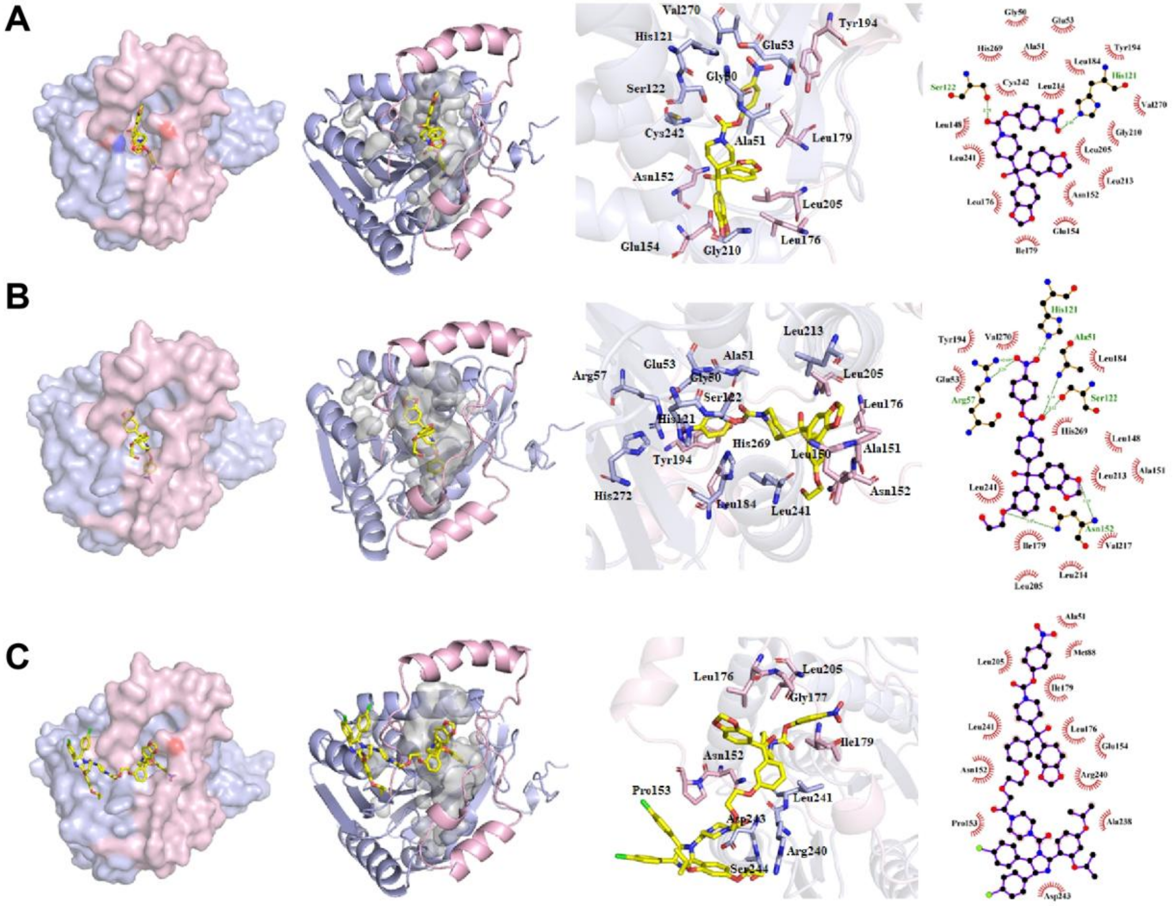

Supplementary Fig. 1 A The surface of MAGL with the JZL184 docked. JZL184 is shown as sticks and the MAGL as cartoon with the cap region in pink and the core in light blue (The first from left). The gray blob demonstrates the cavity inside the protein and the yellow sticks are the docked JZL184 (The second from left). Close up view of the active amino acid residues in the structure of the JZL184 and MAGL complexes. JZL184 is shown as yellow bar and the active amino acid residues are shown in light purple and light pink (The third from left). Ligplot+ graph of JZL184 docked to MAGL. The hydrophobic interactions are shown as brown circle sections and hydrogen bonding interactions are indicated by green dashed lines (Last). **B** Surface view of the MAGL structure with JZL184 analog docked. The MAGL is shown as cartoon and the JZL184 analog as sticks (First from left). The gray spot shows cavities inside proteins and the yellow sticks show docked JZL184 analog (Second from left). Close up view of the active amino acid residues in the structure of the JZL184 analog and MAGL complexes (Third from left). Ligplot+ graph of JZL184 analog docked to MAGL (Last). **C** Surface representation of MAGL with the JN-PROTAC. The MAGL's core in light blue and the cap in pink. The JN-PROTAC is shown as sticks (First from left). The gray blob depicts the cavity inside the protein and the cap in pink, the core in light blue (Second from left). Close up view of the active amino acid residues in the structure of the the JN-PROTAC and MAGL (Third from left). Ligplot+ graph of JZL184 analog docked to the JN-PROTAC (Last).

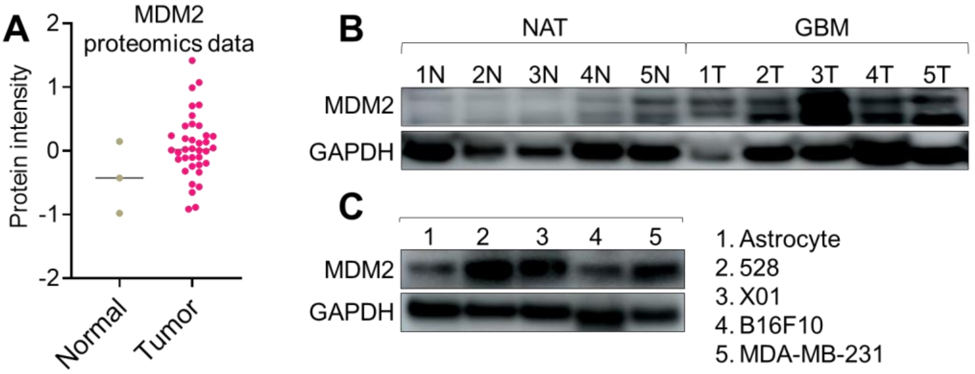

Supplementary Fig. 2 A Protein intensity of MDM2 in normal tissues (Normal) and GBM tumor tissues (Tumor) based on proteomics data. Each dot represents an individual sample (CPTAC GBM proteomics dataset), two-tailed Student's t-test ($p > 0.05$). **B** Immunoblotting for MDM2 expression in paired tumor tissues (GBM) and its normal adjvant tumor tissues (NAT) from five glioblastoma patients. GAPDH was used as a loading control. **C** Immunoblotting for MDM2 expression in non-cancerous human astrocyte cells, human glioblastoma stem cells (528, X01), mouse melanoma (B16F10), and human breast tumor (MDA-MB-231). GAPDH was used as a loading control.

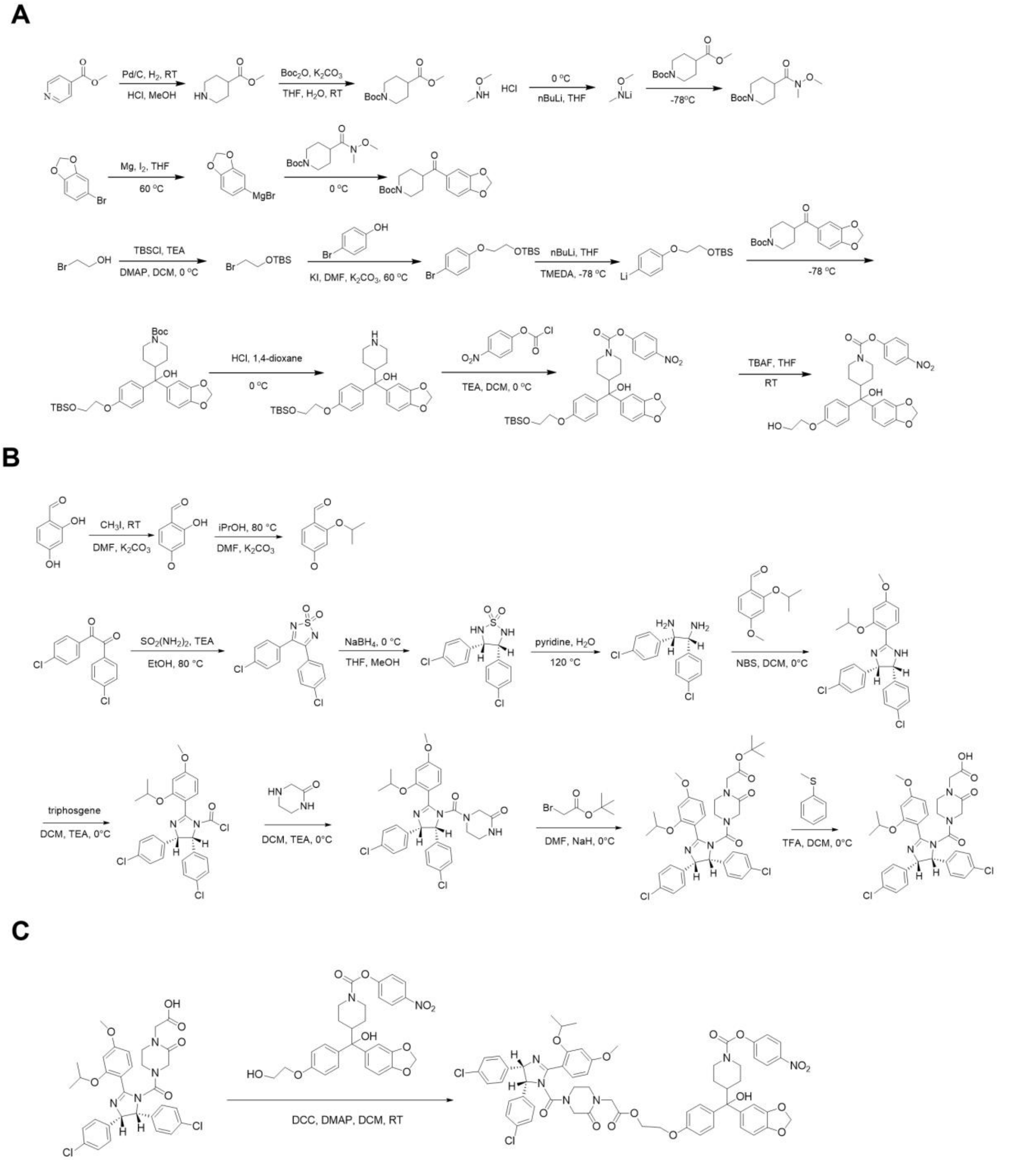

Supplementary Fig. 3 **A** Synthesis scheme for JZL184 analog. **B** Synthesis scheme for Nutlin-3 analog. **C** Synthesis scheme for JN-PROTAC.

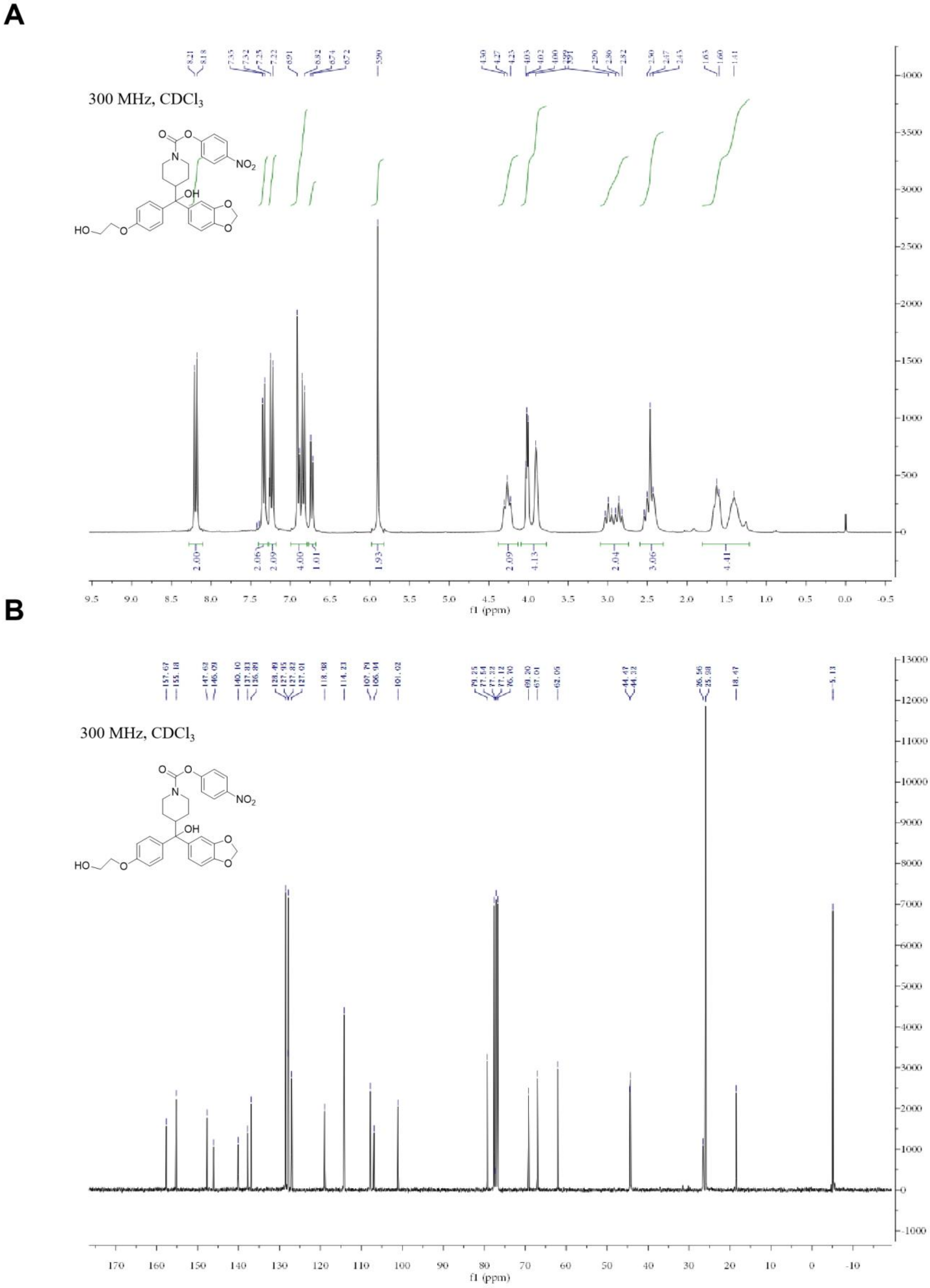

Supplementary Fig. 4 A ^{1}H NMR spectra for JZL184 analog in $CDCl_3$. **B** ^{13}C NMR spectra for JZL184 analog in $CDCl_3$.

A

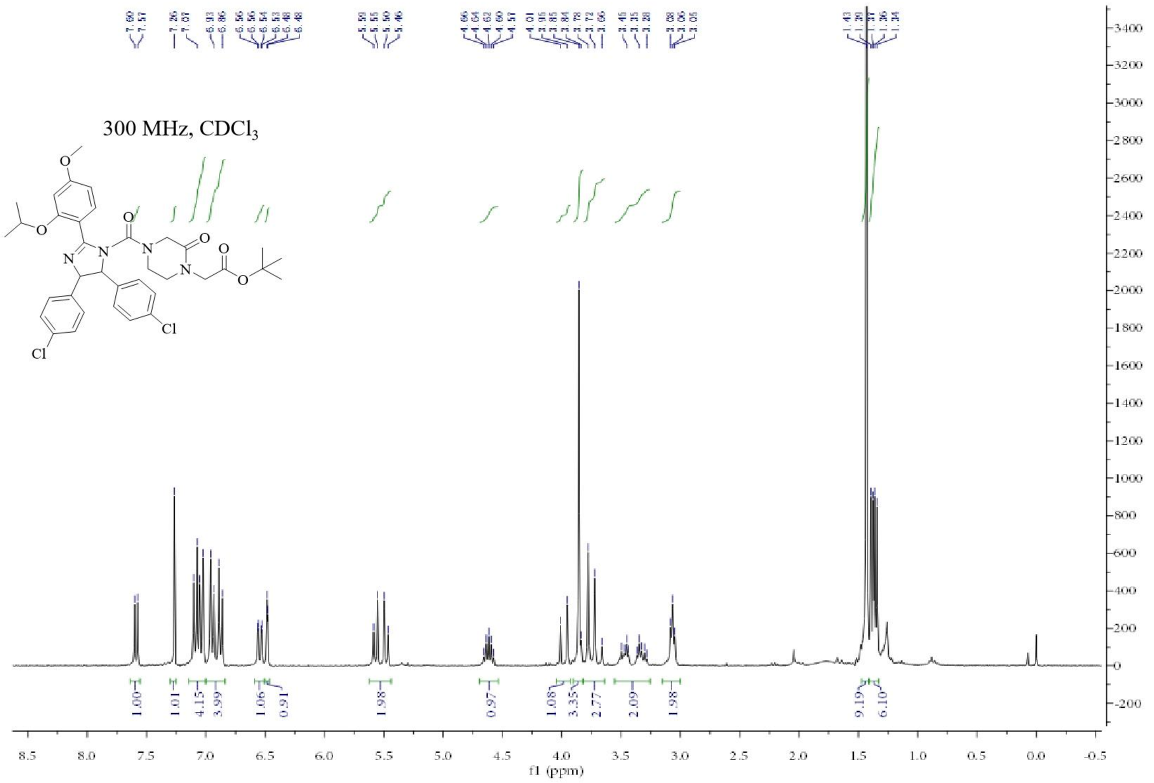

Supplementary Fig. 5 A ^{1}H NMR spectra for Nutlin-3 analog in $CDCl_3$.

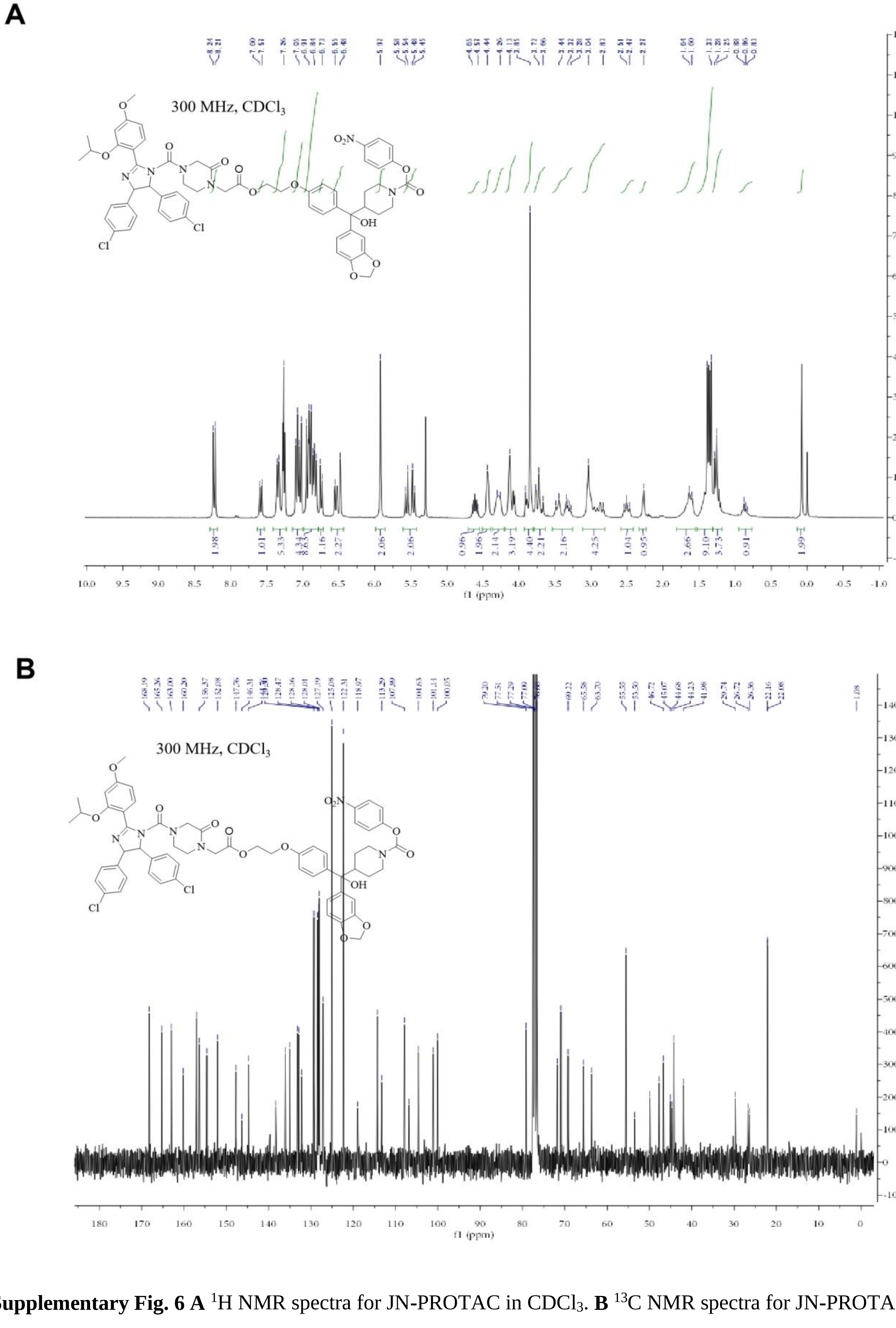

Supplementary Fig. 6 A ^{1}H NMR spectra for JN-PROTAC in $CDCl_3$. **B** ^{13}C NMR spectra for JN-PROTAC analog in $CDCl_3$.

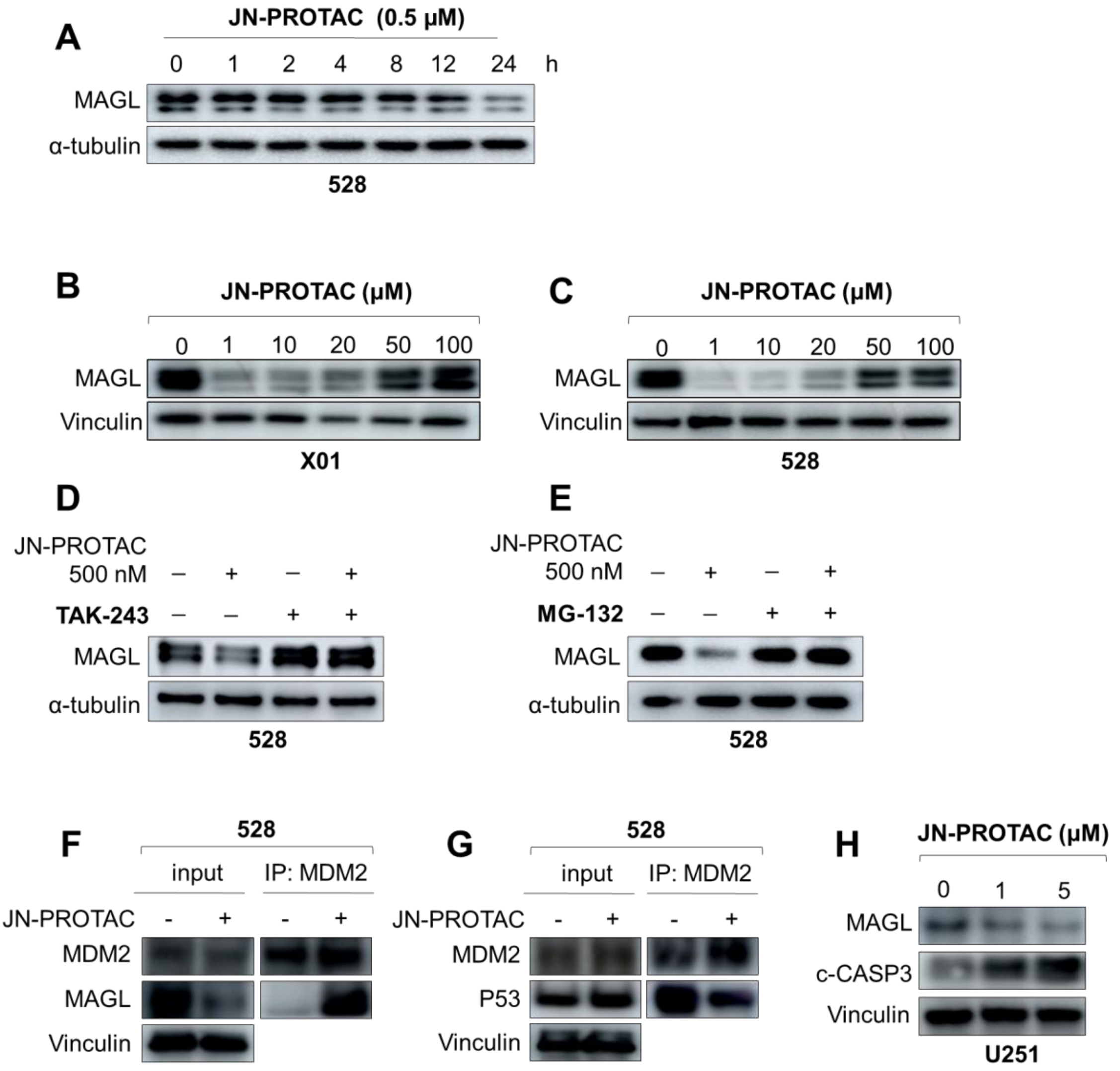

Supplementary Fig. 7 A Immunoblotting for MAGL expression in 528 GSCs treated with 0.5 μM of JN-PROTAC for the indicated durations. α-tubulin was used as a loading control. **B, C** Immunoblotting of MAGL level following 48 h treatment of X01 (B) and 528 GSCs (C) with the indicated concentrations of JN-PROTAC. Vinculin was used as a loading control. **D, E** Immunoblotting for MAGL and MDM2 expression in 528 GSCs after 24 h treatment with 500 nM of JN-PROTAC, 10 μM of TAK-243 (D) or 10 μM of MG-132 (E). α-tubulin was used as a loading control. **F, G** Co-IP assay for the PROTAC-induced ternary complex formation in 528 GSCs treated with JN-PROTAC or DMSO. Cell lysates were precipitated with anti-MDM2 antibody. Vinculin was used as a loading control. Interaction of MDM2 and MAGL (F) and the interaction between MDM2 and P53 (G) in 528 GSCs are presented. **H** Immunoblotting for MAGL and c-CASP3 expression in U251 cells treated with indicated dose of JN-PROTAC. Vinculin was used as a loading control.

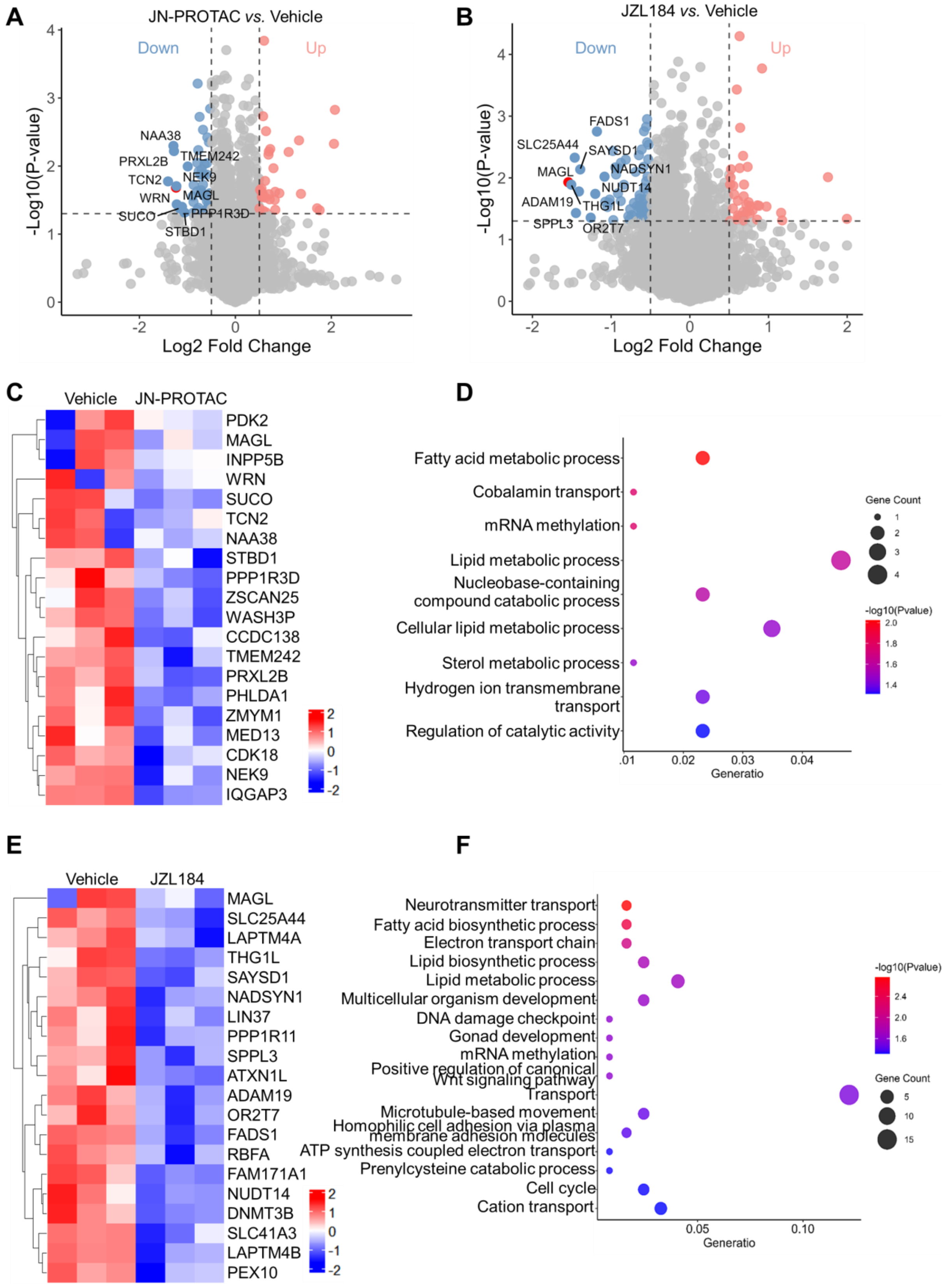

Supplementary Fig. 8 Proteomic and functional enrichment analysis of JN-PROTAC and JZL184. A, B Volcano plots illustrating significantly downregulated and upregulated proteins in the JN-PROTAC (A) and JZL184 (B) treatment groups compared to the DMSO vehicle group in X01 GSCs (48 h, 10 μM). The top 10 downregulated proteins are labeled in each plot, with MAGL highlighted in red. **C** Heatmap showing the top 20 DEPs in X01 GSCs treated with JN-PROTAC compared to the DMSO vehicle group. **D** Gene Ontology (GO) functional enrichment analysis of DEPs in the JN-PROTAC-treated group. **E** Heatmap depicting the top 20 DEPs

in X01 GSCs treated with JZL184 compared to the vehicle group. **F** GO functional enrichment analysis of DEPs in the JZL184-treated group.

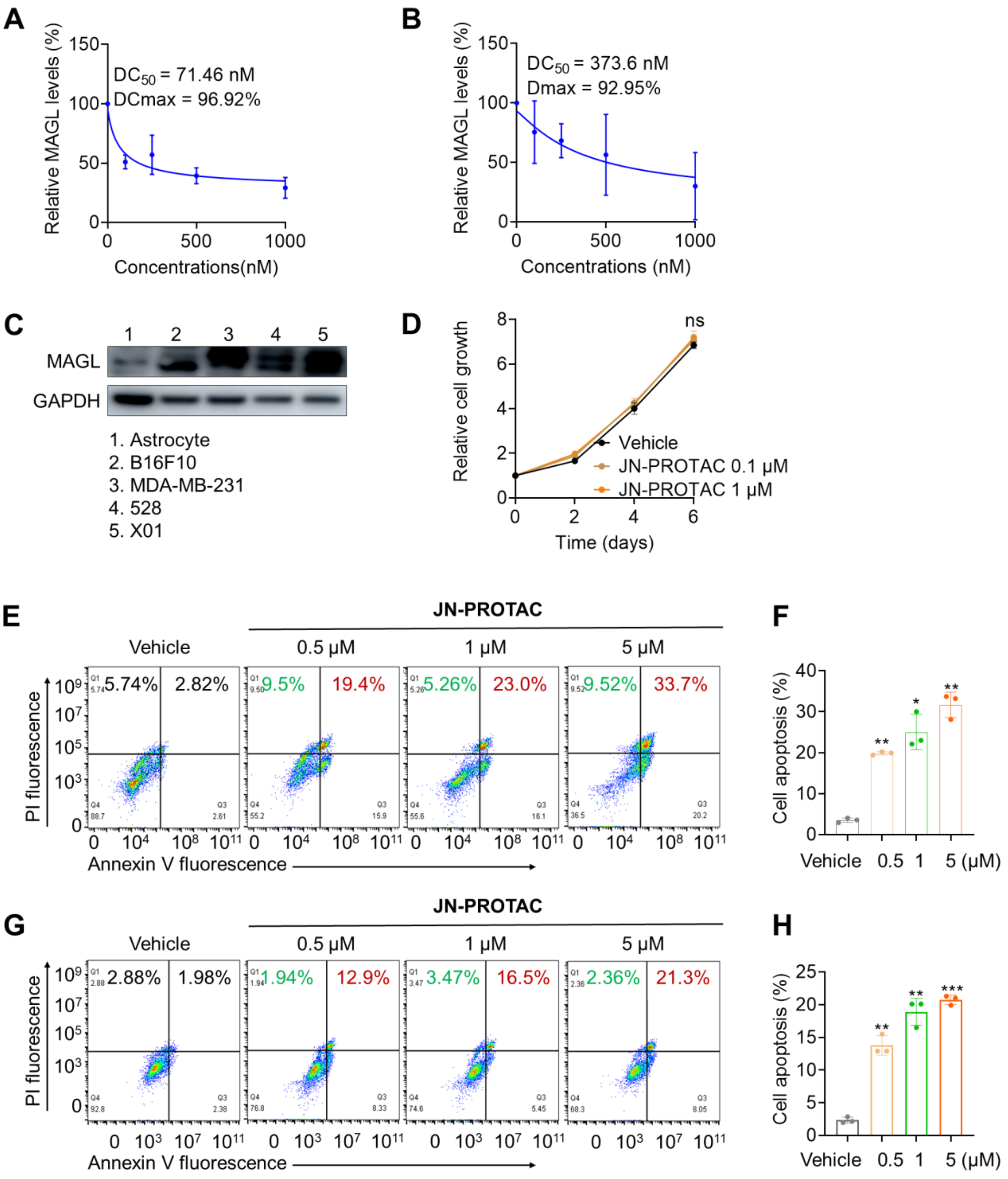

Supplementary Fig. 9 **A** Dose response curves, DC_{50} (The half-maximal degradation concentration), and D_{max} (Maximal degradation %) calculations for JN-PROTAC in 528 GSCs after 24 h by quantitative immunoblotting. **B** Dose response curves, DC_{50} and D_{max} calculations for JN-PROTAC in X01 GSCs after 24 h by quantitative immunoblotting. DC_{50} and D_{max} values are presented as mean ± SD (n = 3, independent experiments). **C** Immunoblotting for MAGL expression in glioblastoma stem cells (528, X01), B16F10, MDA-MB-231 cells, and non-cancerous astrocyte cells. GAPDH was used as a loading control. **D** Cell proliferation assays were performed in astrocytes treated with the indicated concentrations of JN-PROTAC for 6 days. Relative proliferation folds data

are presented as mean ± SD (n = 4, independent experiments), two-tailed Student's t-test (ns $p > 0.05$). **E, G** Flow cytometry for PI/Annexin-V apoptosis assay in X01 (E) and 528 GSCs (G) treated with vehicle or JN-PROTAC (0.5 μM, 1 μM and 5 μM). **F, H** Quantification of total apoptotic cells presented as a percentage of the total cell population at different concentrations of JN-PROTAC (0.5 μM, 1 μM and 5 μM) compared to vehicle. All quantification data are presented as mean ± SD (n = 3, independent experiments), two-tailed Student's t-test (*$p < 0.05$, **$p < 0.01$, ***$p < 0.001$) compared to the vehicle control group.

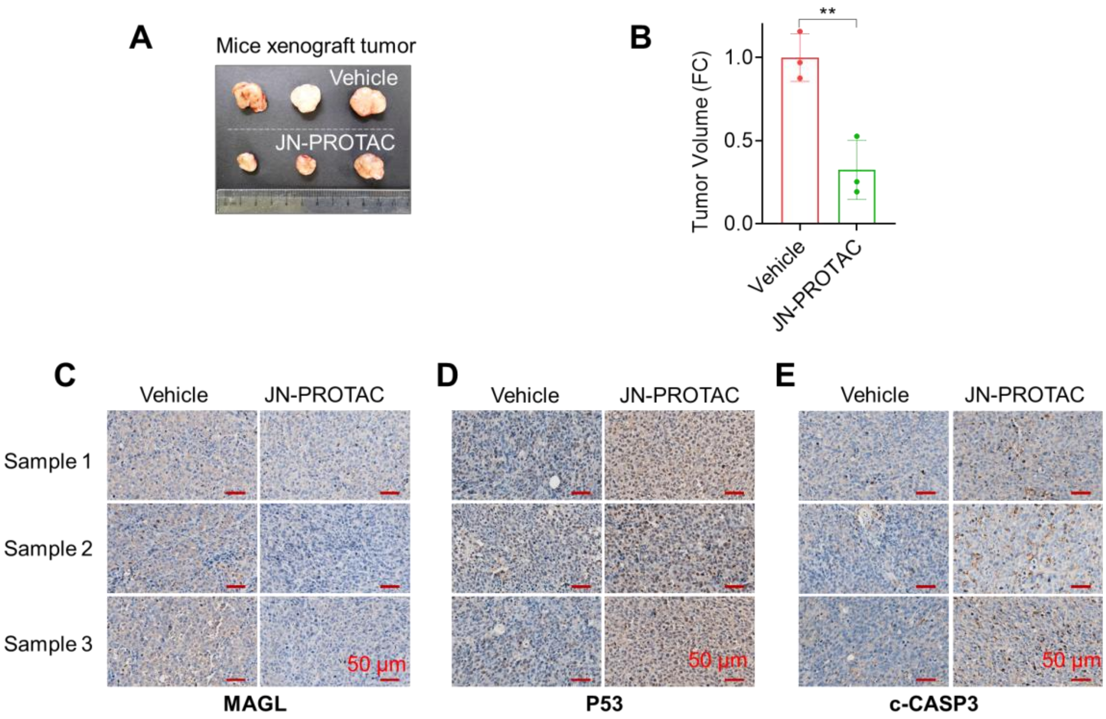

Supplementary Fig. 10 A, B Representative tumor images (A) and quantification (B) for tumor volume fold changes (FC) of X01 GSCs implanted subcutaneous mice treated with JN-PROTAC or vehicle. Data are presented as mean ± SD (n = 3 independent experiments), two-tailed Student's t-test (**$p = 0.0068$). **C-E** Immunohistochemical (IHC) analysis of MAGL (C), P53 (D), and c-CASP3 (E) in the subcutaneous mice model. Scale bar, 50 μm. Samples were collected 24 h after the final drug administration. Refer to Fig. 4.

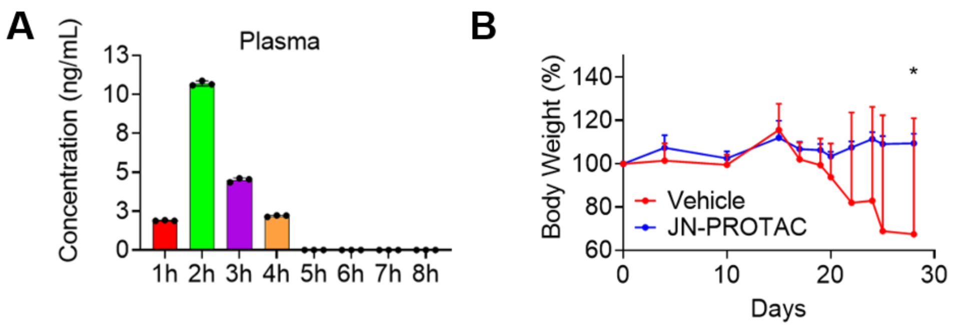

Supplementary Fig. 11 A Plasma accumulation of the JN-PROTAC. Mice were treated with JN-PROTAC at 30 mg/kg orally and serum samples were collected for HPLC analysis of JN-PROTAC at indicated time points. Data are presented as mean ± SD (n = 3, independent experiments). **B** Body weight changes in orthotopic tumor bearing mice model with orally administered JN-PROTAC and non-treatment vehicle. The mice received JN-PROTAC orally (30 mg/kg) five times per week for a consecutive period of 3 weeks (total 15 times). Data are presented as mean ± SD (n = 6, independent experiments), two-tailed Student's t-test (*p = 0.0125).
