# Supplementary material for: MAGL targeted PROTAC degrader simultaneously enhances P53 for synergistic treatment of glioblastoma stem cell
Source: Cell Death Discov. 2025 Mar 20;11:109. doi: 10.1038/s41420-025-02392-1 (PMC11926070; doi:10.1038/s41420-025-02392-1)

Fig. 2A. X01

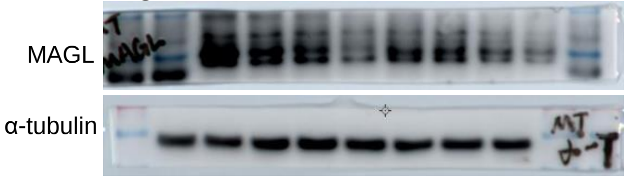

Fig. 2B. X01 JN-PROTAC 0~24h

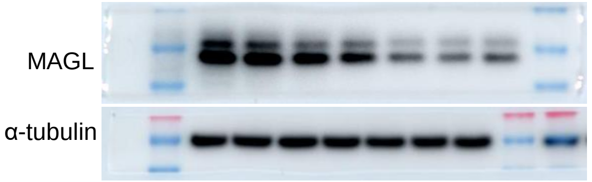

Fig. 2C 528

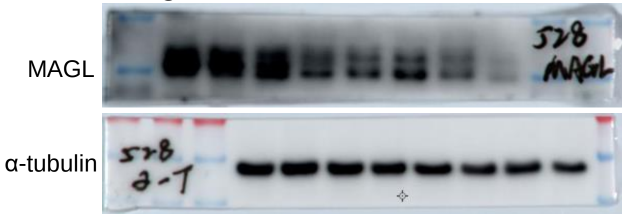

Fig. 2D MDA-MB-231

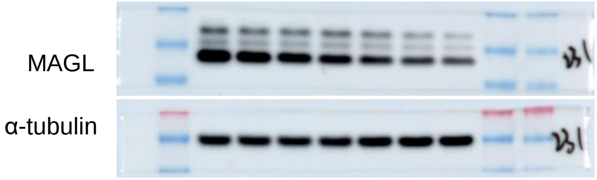

Fig. 2E B16F10

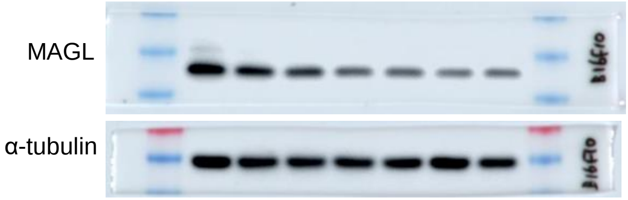

Fig. 2H X01

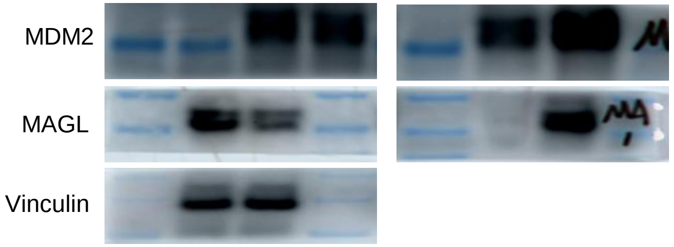

Fig. 2I X01

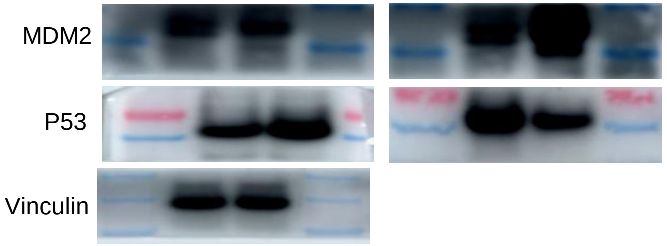

Fig. 2F X01

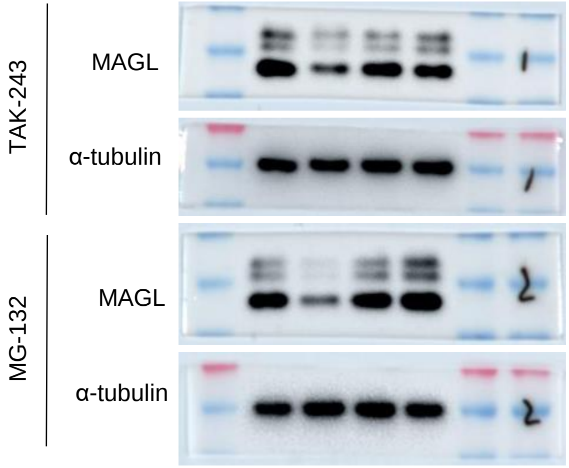

Fig . 2G X01

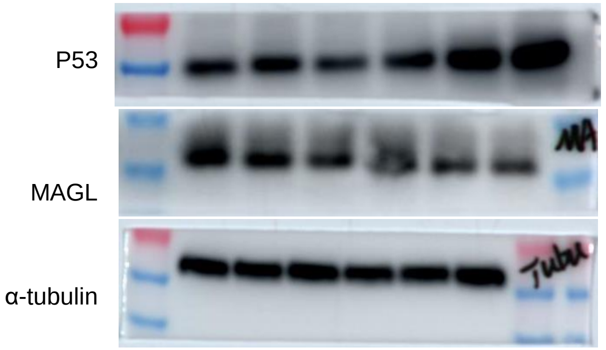

Fig. 3G

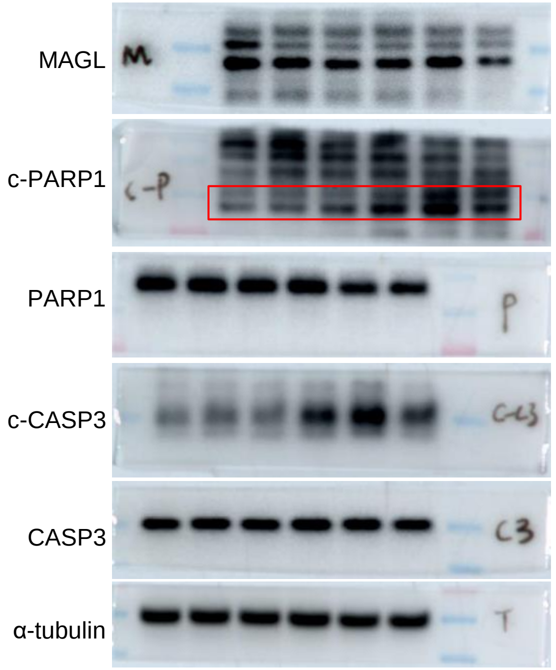
MAGL
c-PARP1
PARP1
c-CASP3
CASP3
α-tubulin

Fig. 3H

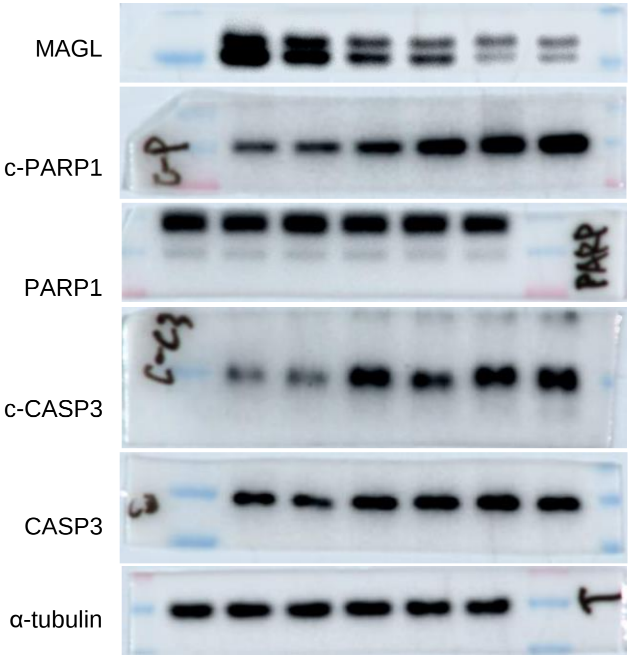
MAGL
c-PARP1
PARP1
c-CASP3
CASP3
α-tubulin

Fig. 4C

MAGL

α-tubulin

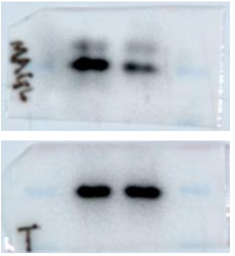

Supplementary Fig. 2B

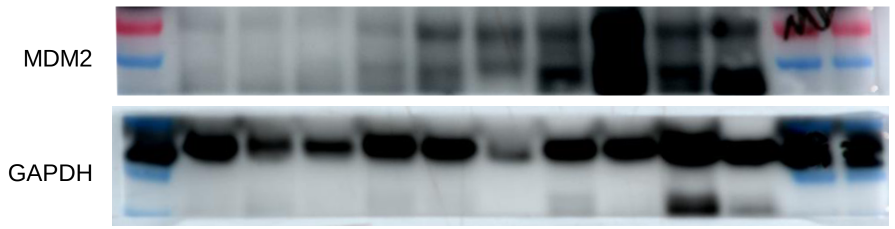

Supplementary Fig. 2C

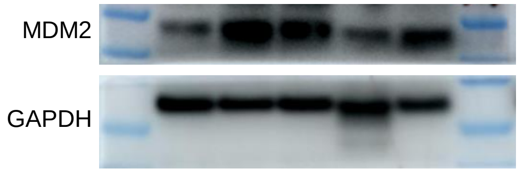

Supplementary Fig. 7A 528 JN-PROTAC 0~24h

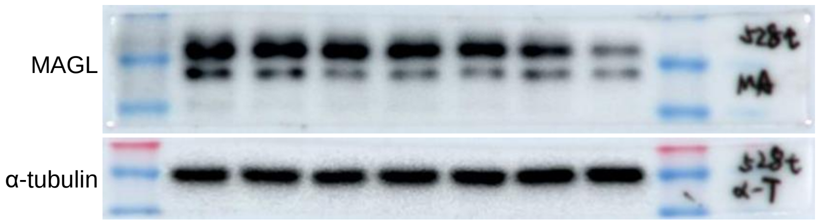

Supplementary Fig. 7B

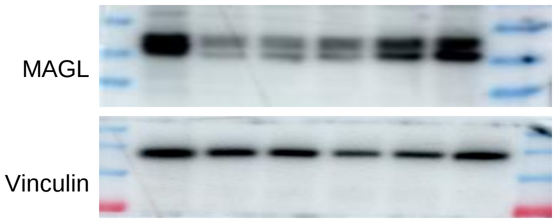

Supplementary Fig. 7C

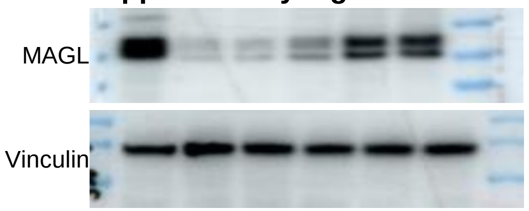

Supplementary Fig. 7D

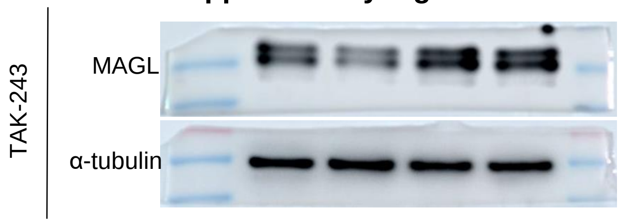

Supplementary Fig. 7E

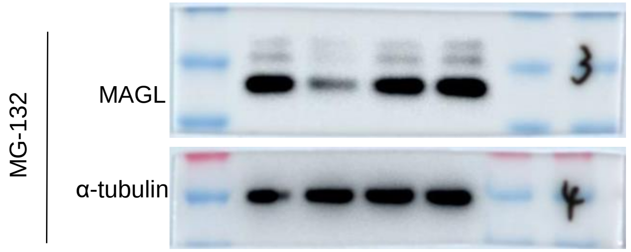

Supplementary Fig. 7F

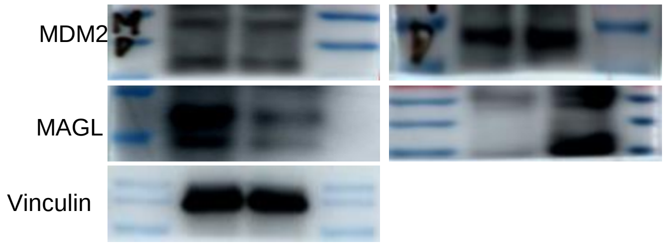

Supplementary Fig. 7G

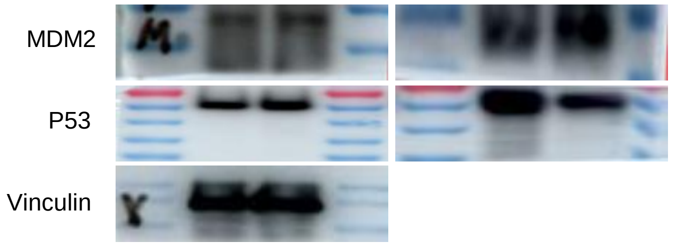

Supplementary Fig. 7H

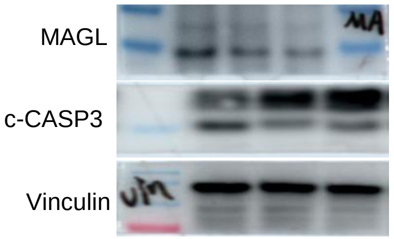

Supplementary Fig. 9C

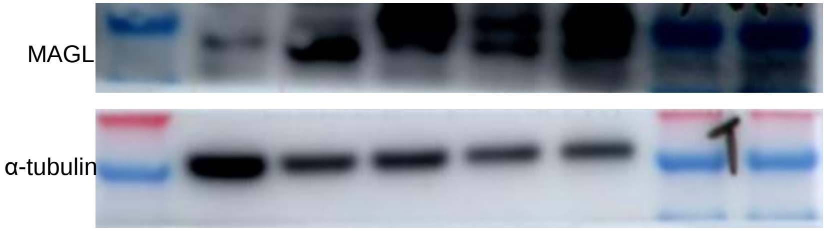

Supplement: Supplementary file 2 — Western blot gels raw data [file 41420_2025_2392_MOESM2_ESM.pdf]
